# Pharmaceuticals and personal care products in Canadian municipal wastewater and biosolids: occurrence, fate, and time trends 2010–2013 to 2022

**DOI:** 10.1007/s11356-025-36007-0

**Published:** 2025-02-03

**Authors:** Sarah B. Gewurtz, Alexandra S. Auyeung, Steven Teslic, Shirley Anne Smyth

**Affiliations:** Science and Technology Branch, Environment and Climate Change Canada, Burlington, ON L7S 1A1 Canada

**Keywords:** Pharmaceuticals and personal care products (PPCPs), Wastewater treatment plants (WWTPs), Temporal changes, Influent and effluent, Biosolids

## Abstract

The concentrations of 135 pharmaceuticals and personal care products (PPCPs) were determined in raw influent, final effluent, and treated biosolids at Canadian wastewater treatment plants (WWTPs) to evaluate the fate of PPCPs through liquid and solids trains of typical treatment types used in Canada and to assess changes in PPCP concentrations in wastewater matrices between 2010–2013 and 2022. PPCPs dominant in influent and effluent included the antidiabetic metformin, analgesics/anti-inflammatories (acetaminophen, ibuprofen, 2-hydroxy-ibuprofen), caffeine and its metabolite (1,7 − dimethylxanthine), theophylline (a bronchodilator and metabolite of caffeine), an insect repellent (N,N-diethyl-m-toluamide, DEET), and iopamidol (a contrast media for X-rays). PPCPs dominant in biosolids differed from those in influent/effluent and included antibiotics (fluoroquinolones and doxycycline), antidepressants (sertraline, citalopram, and amitriptyline), a preservative and antimicrobial agent (triclosan), an antihistamine (diphenhydramine), and an antifungal (clotrimazole). These elevated concentrations in influent/effluent and biosolids reflected their use in Canadian communities. PPCPs dominant in influent/effluent had relatively low hydrophobicity whereas those in biosolids tended to be more hydrophobic, or electrostatic forces governed their sorption. Higher removal of PPCPs was generally observed at WWTPs that used biological treatment compared to primary physical/chemical treatment. PPCP concentration changes in wastewater matrices between 2010–2013 and 2022 were influenced by risk management measures, warnings, the development of new pharmaceuticals, the COVID-19 pandemic, and other factors. These time trends reflected the limited information available on PPCP use in Canada. Continued periodic monitoring of PPCPs is recommended to fill data gaps on community use and release to the environment.

**Supplementary Information:**

The online version contains supplementary material available at 10.1007/s11356-025-36007-0.

## Introduction

Pharmaceuticals and personal care products (PPCPs) consist of a diverse group of substances. Pharmaceuticals are prescription, over-the-counter, and veterinary therapeutic drugs used to prevent or treat human and animal diseases (Boxall et al. 2012; Ebele et al. 2017). Personal care products are substances or mixtures of substances which are used in daily cleansing or grooming (Government of Canada 2008). Some chemicals of this class have been recognized as posing an environmental concern, whereas the environmental risk for others has been deemed low, unclear, or not adequately studied (Boxall et al. 2012; Ambrosio-Albuquerque et al. 2021; Wang et al. 2021). Although some data are available on changing PPCP use patterns over time (Wang et al. 2013; Secrest et al. 2020; Rudnick et al. 2020; Saatchi et al. 2021), this information is incomplete and not available for all PPCPs.

Several previous studies have evaluated PPCPs in municipal wastewater treatment plants (WWTPs) throughout the world (Stasinakis et al. 2013; Guerra et al. 2014; Petrie et al. 2015; Tran et al. 2018; Golovko et al. 2021). These studies have found incomplete and highly variable removal efficiencies of PPCPs between WWTPs (Guerra et al. 2014; Di Marcantonio et al. 2020). The lack of complete removal has been attributed to the fact that WWTPs are not designed to remove chemicals such as PPCPs (Metcalf & Eddy Inc 2003; Tran et al. 2018). Therefore, WWTPs are pathways of these substances to the environment (Thomaidi et al. 2015; Petrie et al. 2015; Verlicchi and Zambello 2015; Golovko et al. 2021). Wastewater influent can provide information on community usage of PPCPs (Kasprzyk-Hordern et al. 2021; Boogaerts et al. 2021; Duan et al. 2022). For example, a literature review of PPCPs across Asia, Europe, and North America found large variations in PPCP concentrations in influent that were hypothesized to be due in part to differences in usage patterns between regions (Tran et al. 2018). In addition, several studies have used wastewater-influent data to indicate pharmaceutical use changes due to the COVID-19 pandemic (Boogaerts et al. 2021; Luo et al. 2023; Ting et al. 2024). PPCP concentrations in wastewater effluent and biosolids vary widely between WWTPs and are influenced by treatment type and PPCP properties such as the potential to sorb onto solids, volatility, and susceptibility to transformation processes, in addition to the PPCP input received by the WWTPs (Guerra et al. 2014; Petrie et al. 2015; Tran et al. 2018; Bavumiragira et al. 2022). PPCPs in effluent and biosolids provide information on PPCPs being released into the environment (Thomaidi et al. 2015; Mejías et al. 2021; Bavumiragira et al. 2022). Although data from WWTPs have been recognized as tools to assess changes in usage and release to the environment (Tran et al. 2018; Boogaerts et al. 2021; Luo et al. 2023), large-scale country-wide studies to assess changes over time are lacking.

In Canada, Guerra et al. (2014, 2019) studied PPCPs in six treatment plants between 2010 and 2013. In 2022, we had the opportunity to conduct additional analysis of PPCPs in Canadian WWTPs as part of a long-term monitoring program. This provided an opportunity to assess country-wide changes in PPCP concentrations in wastewater matrices over a nearly 10-year time period. This was needed because there was limited information on PPCP use and release into the Canadian environment. The first objective of this study was to assess concentrations of 135 PPCPs in raw influent, final effluent, and treated biosolids collected from seven representative Canadian WWTPs in 2022. The second objective was to evaluate the removal of PPCPs within different wastewater treatment types. The third objective was to evaluate whether PPCP concentrations have changed in wastewater matrices over time by comparing data for samples collected in 2022 with those collected between 2010 and 2013. This study provides information that is critical for identifying factors that have resulted in changes to PPCP usage and/or the concentrations of PPCPs being released into the environment. In addition, this study provides useful information to feed into assessments and for determining the need for risk management.

## Methods

### Sample collection

In total, this study included data from 32 WWTP sampling events (Table S1.1 of Supplementary Information (SI) 1). Seven WWTPs were included in the 2022 sampling campaign for PPCPs (Table S1.1). Additionally, ten WWTPs were sampled at least once between 2010 and 2013 (Table S1.1). Influent and effluent were collected from every WWTP visited. Biosolids were collected from the WWTPs that generated them (Table S1.1). These WWTPs were selected to represent typical Canadian treatment systems and geographic variations (Guerra et al. 2014; Gewurtz et al. 2022, 2024). The WWTPs participated in this program on the condition of anonymity and are referred to by codes. WWTP characteristics, sampling dates, and water temperatures for each WWTP and sampling event are provided in Table S1.1.

Each WWTP sampling event consisted of the collection of raw influent, final effluent, and treated biosolids on three consecutive weekdays. Twenty-four-hour equal-volume composite samples of influent and effluent (between 2010 and 2013, 400 mL every 30 min; 2022, 200 mL every 15 min) were collected at most plants (Table S1.1). However, grab samples were necessary for influent and/or effluent at some WWTPs because the flow characteristics and access were not amenable to composite sampler installation, or due to composite sampler malfunction (Table S1.1). Influent and effluent samples were collected at the same time with no compensation for hydraulic retention time. Biosolids samples were collected as grabs. The sludge collection and treatment processes inherently composited the solids due to the retention time in clarifiers and digesters. Process temperature was measured every day samples were collected. The measured average daily flows (m^3^/days) and process temperatures for each WWTP are listed in Table S1.1.

Composite samples were collected using refrigerated autosamplers (HACH Company, Loveland, CO, USA). Following collection, the composite samples were poured into 18-L stainless steel canisters that had been cleaned with Contrad 70 detergent (Decon Laboratories, Inc., Bryn Mawr, PA, USA) and rinsed sequentially with methanol (reagent grade, Caledon Laboratories, Georgetown, Canada) and deionized water prior to use. Grab samples of bulk biosolids were collected into 10-L stainless steel pails that had undergone the same cleaning procedure as those used for influent and effluent. Bulk raw influent, final effluent, and biosolids samples were subdivided into Nalgene™ high-density polyethylene bottles (Thermo Fisher Scientific, Waltham, MA, USA). Samples were packed in coolers with natural ice and shipped by overnight courier to the laboratories or stored at 4 °C prior to arrival at the laboratories.

Concentrations of antibiotics, analgesic/anti-inflammatories, and antimicrobials measured between 2010 and 2013 were reported previously (Guerra et al. 2014, 2019). All samples were collected by the same team and temporal trend analyses were conducted on data generated by the same analytical laboratory to optimize consistency over time.

### Analytical methods

Chemical analysis, instrumental analysis, and quality assurance and quality control (QA/QC) methods and results are presented in Table S1.2 to S1.16 and described in SI 2. In summary, 135 PPCPs were analyzed by SGS AXYS Analytical Services Limited (SGS AXYS) according to SGS AXYS in-house method MLA-075 (Table S1.2). This method was based on USEPA method 1694 (USEPA 2007). SGS AXYS is accredited to ISO/IEC 17025:2017 standards by the Canadian Association for Laboratory Accreditation (CALA). SGS AXYS has participated in numerous round-robins and intercalibration studies where its proficiency in PPCP analysis was demonstrated. Wastewater influent and effluent samples were filtered prior to extraction and the dissolved phase of these samples was analyzed during both time periods.

The samples of influent, effluent, and biosolid were analyzed for conventional wastewater parameters at Environment and Climate Change Canada (ECCC)’s National Laboratory for Environmental Testing (NLET) according to methods from the American Public Health Association (2012). Conventional wastewater parameter results are described in SI 2 and raw data are available at Government of Canada (2025). Based on these data, we determined that the WWTPs included in this study were receiving low-, medium-, and high-strength wastewater and achieving typical removals of chemical oxygen demand and total suspended solids for each treatment type (Metcalf & Eddy Inc 2003). This indicates that the WWTPs were operating normally during the sampling periods (Metcalf & Eddy Inc 2003).

### Data analysis

Statistical analysis was conducted using R Version 4.3.1 and the methods were chosen for their ability to handle censored measurements (Helsel 2012). At each WWTP and sampling event, the median of PPCP concentrations measured on three consecutive days was used for the tests. Statistical differences in PPCP concentrations in influent and biosolids between WWTPs as well as comparison of removals of PPCPs between treatment types were assessed using the non-parametric Peto-Peto one-factor test of differences in cumulative distribution functions between groups, as recommended by Helsel (2012). Unplanned multiple comparisons were also performed with the Peto-Peto one-factor test (Helsel 2012). The Peto-Peto one-factor test was implemented with the *cen1way* function of the *NADA2* package (Julian and Helsel 2023). Tests were performed on PPCPs with greater than 20% detection frequency. The percent removal datasets contained left and right interval censored data; specifically, there were percent removal values calculated for situations when the analyte was detected in effluent and not influent and vice versa. Therefore, we used the *Usci* function of the *NADA2* package, which incorporates multiply censored datasets (Julian and Helsel 2023) to rank the percent removal data prior to conducting the Peto-Peto one-factor test.

The Peto-Peto one-factor test (Helsel 2012; Julian and Helsel 2023) was also used to test for significant differences in PPCP concentrations in influent, effluent, and biosolids between the two time groups, 2010–2013 and 2022. Time trends of PPCPs were evaluated across all WWTPs to provide an indication of the overall time trends of PPCPs in Canadian WWTPs. For a given matrix, this statistical test was performed on PPCPs with at least two WWTP-specific medians above the detection limit.

Kendall’s tau correlation was used to test for the relationship between median influent concentration and population (Helsel 2012). For each analyte, the median concentration measured on three consecutive days was used in the regression. This test was implemented with the *cenken* function of the *NADA* package (Lee 2020).

To evaluate the relative partitioning of contaminants to biosolids compared to effluent, solid–liquid distribution coefficients (*K*_d_) were experimentally calculated by dividing PPCP concentrations in biosolids (ng/kg) by their respective concentrations in effluent (ng/L) (Guerra et al. 2014). This calculation was influenced by factors including the transformation of PPCPs occurring during sludge treatment as well as temperature, pH, sludge type, and reactor configuration (Stasinakis et al. 2013; Verlicchi and Zambello 2015; Tran et al. 2018). However, the calculation provided an indication of general chemical characteristics and information on whether environmental release occurred primarily through effluent or biosolids for each PPCP.

Removal of PPCPs from wastewater treatment was calculated as ((Influent–Effluent)/Influent) expressed as a percentage. Previous studies have calculated PPCP removal from WWTPs using influent and effluent concentrations (Guerra et al. 2014; Ashfaq et al. 2017; Golovko et al. 2021; Dubey et al. 2022) or loads (Stasinakis et al. 2013; Subedi et al. 2017; Thiebault et al. 2017) of PPCPs. In this study, we assumed steady-state conditions and calculated the removal of PPCPs from the WWTPs using influent and effluent concentrations. Under steady-state conditions, on a given sampling date, the average daily flow of influent at a given WWTP is equal to the flow of effluent (von Sperling et al. 2020). Under these conditions, PPCP removal from WWTPs calculated with influent and effluent concentrations is equal to removal calculated with pollutant loads. Only measured PPCPs were considered as there was no information available on unmeasured precursor, transformation, or breakdown substances. If the substance was detected in influent but not in effluent, we used the detection limit PPCP concentration in effluent to estimate a removal value, and the resulting calculation represented the lower bound of percent removal for the data point, with the upper bound being 100%. If the substance was detected in effluent but not in influent, we used the detection limit PPCP concentration in influent to estimate a removal value, and the resulting calculation represented the upper bound of percent removal for the data point. We did not calculate removal when a PPCP was not detected in both influent and effluent. Kendall’s tau correlation was used to test for the relationship between median percent removal and median log *K*_d_.

## Results and discussion

### PPCPs in wastewater influent

**Fig. 1:**
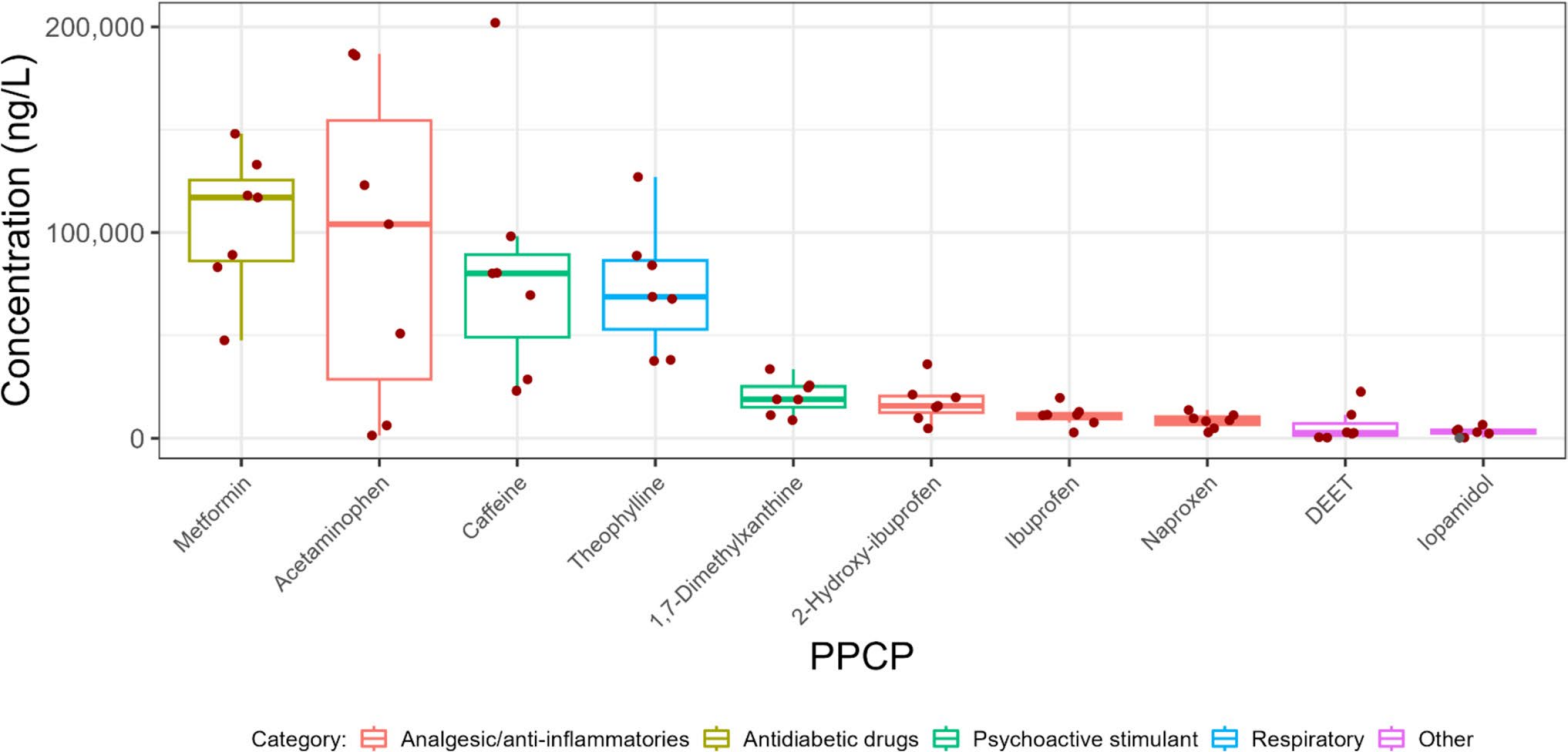
Influent concentrations of PPCPs with the ten highest median values of those measured in this study in 2022. The data points used in this figure represent the median PPCP concentration measured on three consecutive days at each WWTP and sampling event. The line within the boxes shows median concentrations, the boxes show 25th and 75th percentiles, and the whiskers below and above the boxes show 10th and 90th percentiles. Dots show individual data points. Red dots show detected concentrations and grey dots show concentrations below detection and plotted at the detection limit

The minimum, median, and maximum concentrations as well as detection frequencies of PPCPs in wastewater influent are shown in Table S1.20. PPCPs with the ten highest median concentrations in influent in 2022 are shown in Fig. 1. These ten PPCPs had 100% detection frequency, with the exception of iopamidol which had 71% detection frequency (Table S1.20). Dominant PPCPs in this study were comparable to those observed previously in Canada (Guerra et al. 2014 and Table S1.20) and throughout the world (Tran et al. 2018; Golovko et al. 2021; Tomsone et al. 2024) although the PPCP analyte lists were not identical. The median concentration of metformin, an antidiabetic, of 117,000 ng/L was the highest among the substances evaluated in this study. Metformin is widely used in Canada and throughout the world (Ramzan et al. 2019; Secrest et al. 2020; Littlejohn et al. 2023) and is detected globally in WWTP influent as well as in effluent, surface water, drinking water, and groundwater (He et al. 2022). Previously studies have found metformin concentrations in influent ranging from 1421 to 325,000 ng/L (He et al. 2022), which overlapped the range of metformin concentrations observed in this study of 41,700–155,000 ng/L. Four PPCPs in the analgesic/anti-inflammatories category were present at relatively high concentrations in Canadian wastewater: acetaminophen (median = 104,000 ng/L), ibuprofen (median = 11,200 ng/L), 2-hydroxy-ibuprofen (an ibuprofen metabolite) (median = 16,000 ng/L), and naproxen (median = 8390 ng/L). Similar results in Canada were reported previously by Guerra et al. (2014) (see also data presented in Table S1.20). The concentrations of these analgesic/anti-inflammatories fell within the range observed previously for these substances in Asia, North America, and Europe (Aymerich et al. 2016; Tran et al. 2018). These substances are easily accessible without a prescription and are widely used by consumers (Kim et al. 2022). Caffeine and its metabolite (1,7 − dimethylxanthine) were present in Canadian wastewater influent at relatively high concentrations (median = 71,200 and 20,900 ng/L, respectively), which was not surprising considering the popularity of coffee and other caffeine-containing food and beverages (Korekar et al. 2020). Similar concentrations of caffeine were found in Canada between 2010 and 2013 (Table S1.20) and in North America and Europe although concentrations of caffeine in Asia were slightly lower (range = 759–60,600 ng/L) (Tran et al. 2018). Theophylline was present at similar concentrations as caffeine (median = 79,800 ng/L). In comparison, concentrations of theophylline in influent of 15 WWTPs located in Sweden in 2018 (1400–2800 ng/L) (Golovko et al. 2021) and in five WWTPs in Malaysia in 2015 (71–319 ng/L) (Al-Qaim et al. 2018) were lower than those observed in this study (range = 30,600–141,000 ng/L). Theophylline is a bronchodilator and is used to treat chronic obstructive pulmonary disease and asthma (Montaño et al. 2022; Boylan et al. 2023). Theophylline is also effective in the treatment of COVID-19 symptoms (Montaño et al. 2022). It is noteworthy that influent concentrations in WWTPs sampled prior to the onset of the COVID-19 pandemic (Al-Qaim et al. 2018; Golovko et al. 2021) as well as in Canadian WWTPs sampled between 2010 and 2013 (Table S1.20) were lower than those observed in this current study in 2022; this is discussed below. Additionally, theophylline is a minor metabolite (7–8%) of caffeine (Lelo et al. 1986) and conversion of theophylline to caffeine can also occur (Haley 1983). N,N-diethyl-m-toluamide (DEET), an insect repellent, and iopamidol, which is used as a contrast media for X-rays, were also present at relatively high concentrations in wastewater influent (median = 2880 and 2980 ng/L, respectively); these concentrations were comparable to those observed in wastewater influent collected from Asia, North America, and Europe (Tran et al. 2018).

**Fig. 2:**
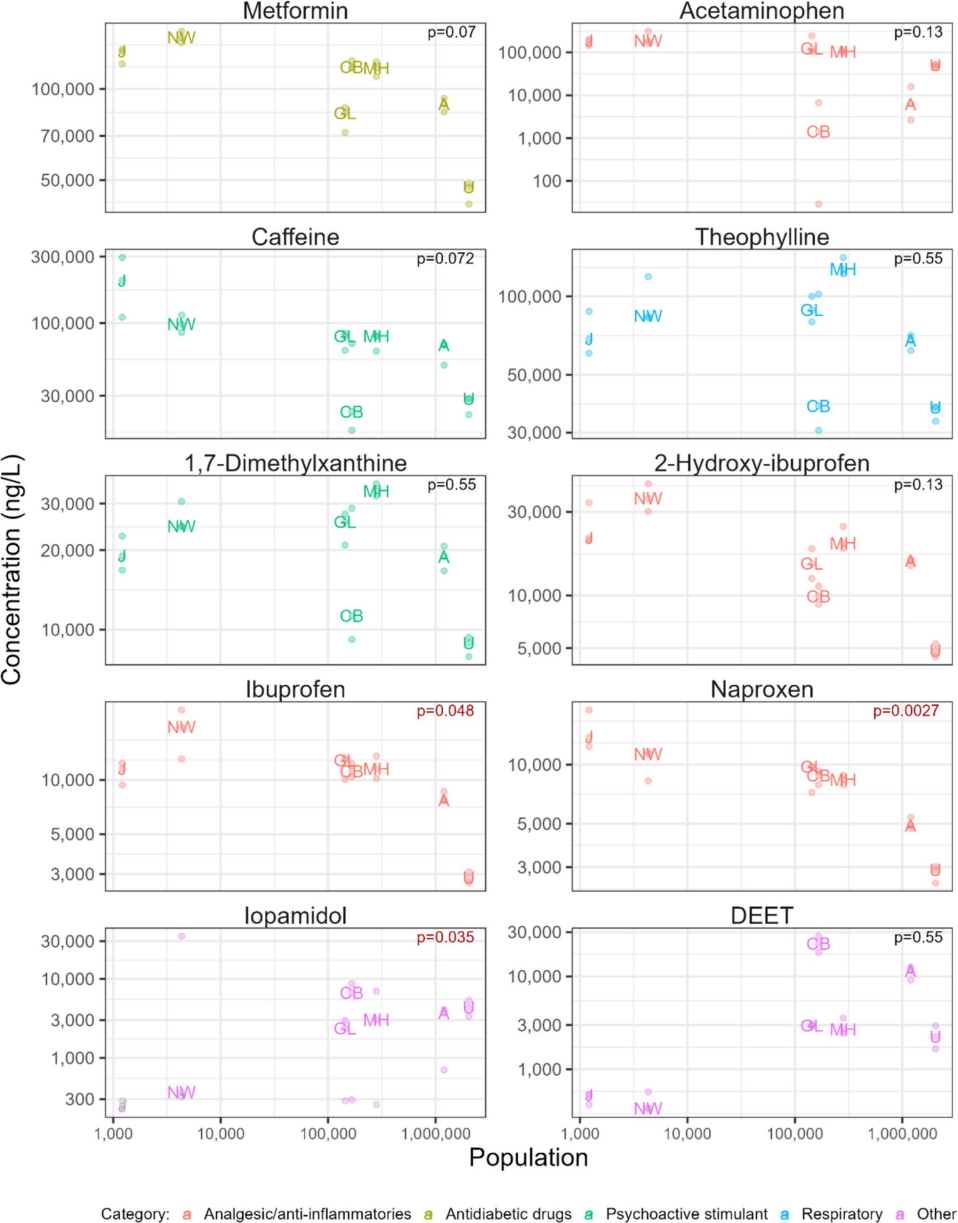
Median concentrations of representative PPCPs in wastewater influent as a function of population served in 2022. WWTP codes indicate median influent concentrations. Circles indicate individual data points; colored circles = detected concentrations, grey circles = concentrations below detection and plotted at the detection limit. The *p*-values indicate the significance of Kendall’s correlation coefficient, with *p*-values in red and black font indicating significant (*p* < 0.05) and not significant (*p* > 0.05) relationships, respectively. Note the log scale

Per-capita consumption of PPCPs can be estimated as the product of the influent PPCP concentration and the influent flow (PPCP mass load), divided by the population served by the WWTPs (O’Brien et al. 2014; Gao et al. 2016, 2023). In the present study, PPCP concentrations of most chemicals in influent differed significantly between WWTPs (*p* < 0.05) but there were mostly no significant relationships (*p* > 0.05) between influent concentrations and the size of the population served by the WWTPs (Fig. 2 and Fig. S2.1a-g). Given that WWTP flow and population served were significantly related (*p* < 0.001, *r*^2^ = 0.99), this indicates that the per-capita consumption of PPCPs across WWTPs was mostly independent of the size of the connected population. However, for some PPCPs, there was a trend of significantly (*p* < 0.05) elevated concentrations in the influent of WWTP J. This could be because 100% of the flow at WWTP J was from residential inputs. In addition, a retirement home was present in this community. Concentrations of several PPCPs were relatively low in the influent of WWTP U. According to the operator of WWTP U, this plant received some infiltration during both wet and dry weather due to the age and leakage of the collection system which diluted all wastewater constituents. Previously, multiple factors were hypothesized to affect per-capita consumption of PPCPs such as socio-economical characteristics of the population, life style, population health status, and location-specific prescription availability and price (O’Brien et al. 2014; Gao et al. 2016).

### PPCPs in wastewater effluent

**Fig. 3:**
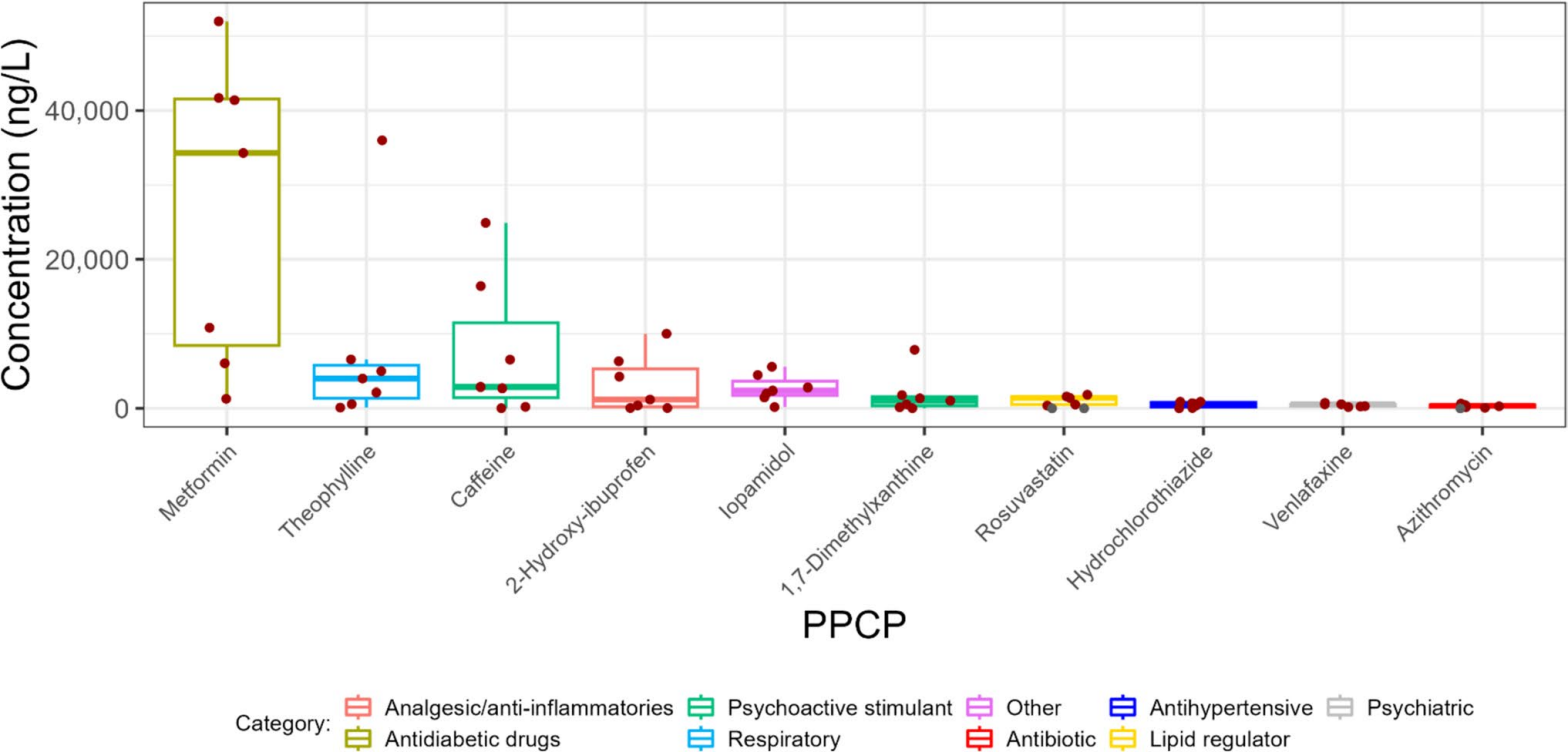
Effluent concentrations of PPCPs with the ten highest median values of those measured in this study in 2022. The data points used in this figure represent the median PPCP concentration measured on three consecutive days at each WWTP and sampling event. The line within the boxes shows median concentrations, the boxes show 25th and 75th percentiles, and the whiskers below and above the boxes show 10th and 90th percentiles. Dots show individual data points. Red dots show detected concentrations and grey dots show concentrations below detection and plotted at the detection limit

Minimum, median, and maximum concentrations as well as detection frequencies of PPCPs in wastewater effluent are shown in Table S1-21. PPCPs with the ten highest median effluent concentrations in 2022 are shown in Fig. 3. These ten PPCPs were detected in 100% of effluent samples, except for azithromycin and rosuvastatin, which had detection frequencies of 86% and 76%, respectively (Table S1.21). PPCPs with elevated concentrations in effluent were comparable to influent. Metformin had elevated concentrations compared to other PPCPs (median = 34,300 ng/L), which was within the range of metformin concentrations previously found in WWTP effluent throughout the world (He et al. 2022). Other dominant PPCPs in effluent included theophylline (median = 3740 ng/L), caffeine (median = 2860 ng/L) and its metabolite (1,7 − dimethylxanthine) (median = 973 ng/L), 2-hydroxy-ibuprofen (median = 1190 ng/L), iopamidol (median = 2650 ng/L), rosuvastatin (a frequently prescribed lipid regulator) (median = 518 ng/L), hydrochlorothiazide (an antihypertensive) (median = 584 ng/L), venlafaxine (a serotonin-norepinephrine reuptake inhibitor) (median = 535 ng/L), and azithromycin (an antibiotic) (median = 331 ng/L). Except for theophylline, concentrations of these substances were comparable with those observed previously in WWTP effluent in Asia, North America, and Europe (Kostich et al. 2014; Guerra et al. 2014; Golovko et al. 2014, 2021; Aymerich et al. 2016; Tran et al. 2018). Similar to influent, theophylline concentrations observed in WWTP effluent collected prior to the start of the COVID-19 pandemic in early 2020 were generally below those observed in this study (Kostich et al. 2014; Petrie et al. 2015; Golovko et al. 2021).

### PPCPs in biosolids

**Fig. 4:**
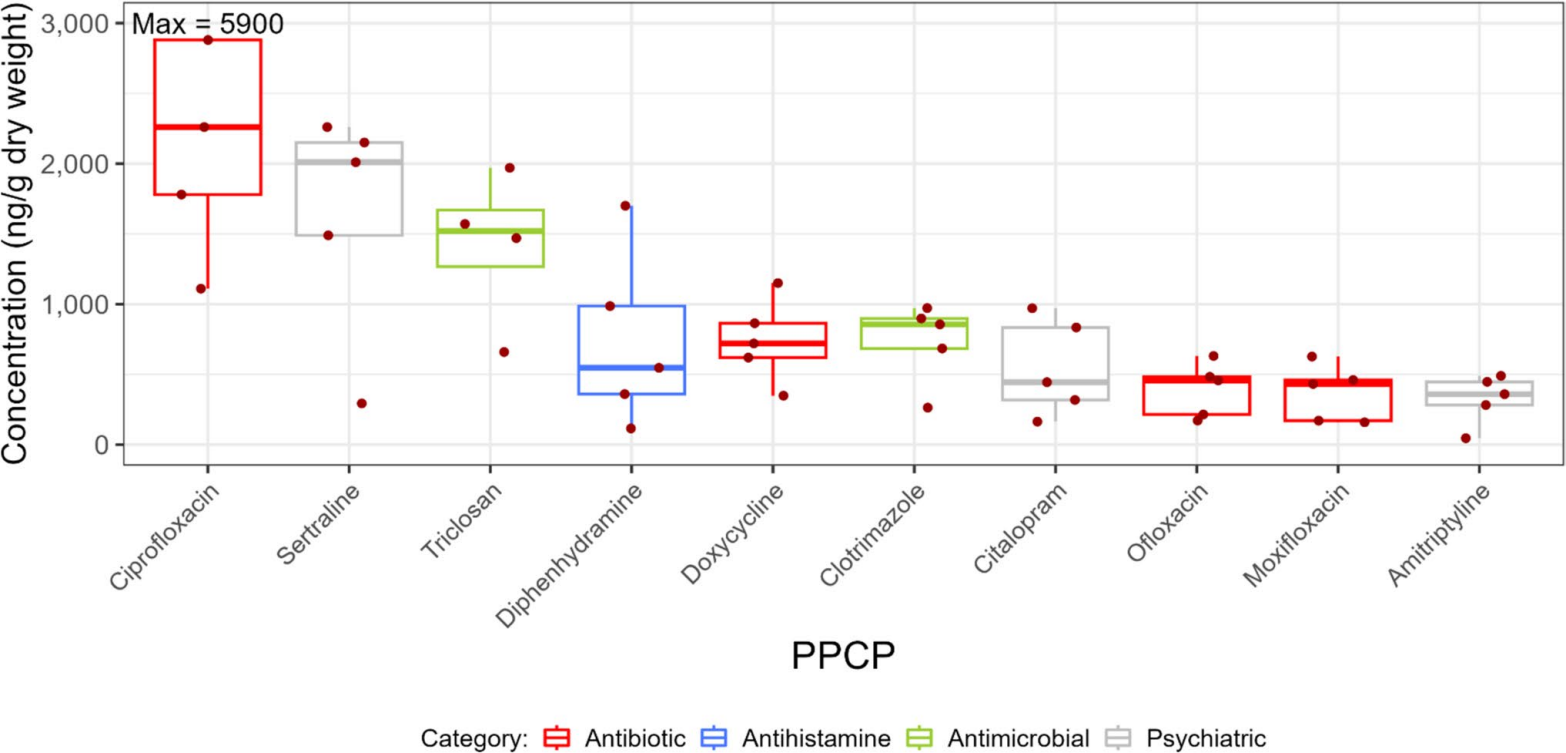
Biosolid concentrations of PPCPs with the ten highest median values of those measured in this study in 2022. The data points used in this figure represent the median PPCP concentration measured on three consecutive days at each WWTP and sampling event. The line within the boxes indicates median concentrations, the boxes show 25th and 75th percentiles, and the whiskers below and above the boxes show 10th and 90th percentiles. Dots show individual data points. Red dots show detected concentrations and grey dots show concentrations below detection and plotted at the detection limit. The dot representing the maximum concentration of ciprofloxacin of 5900 ng/g dry weight is not shown

Minimum, median, and maximum concentrations as well as detection frequencies of PPCPs in biosolids are shown in Table S1-22. PPCPs with the ten highest median biosolids concentrations in 2022 are shown in Fig. 4. These ten PPCPs were detected in 100% of biosolids samples. Dominant PPCPs in biosolids included ciprofloxacin (median = 2260 ng/g), ofloxacin (median = 457 ng/g), and moxifloxacin (median = 432 ng/g), which are fluoroquinolone antibiotics used to treat bacterial infections (Crain et al. 2017; Rudnick et al. 2020). These levels were within the range of concentrations observed previously in biosolids (Verlicchi and Zambello 2015; Tran et al. 2018). Sertraline and citalopram, which are frequently prescribed antidepressants in the selective serotonin reuptake inhibitors (SSRIs) class (Do et al. 2016; Morkem et al. 2017; Luo et al. 2020), were also present at high concentrations (median = 2010 and 444 ng/g, respectively). Sertraline exhibits antiviral activity against SARS-CoV-2 and helps alleviate symptoms associated with infection (Chen et al. 2022). Sertraline concentrations in dewatered and biologically digested sludge collected prior to the onset of the COVID-19 pandemic (170–913 ng/g) (Verlicchi and Zambello 2015; Golovko et al. 2021) were less than those observed in this study. Other dominant PPCPs included the following: triclosan (median = 1545 ng/g), a commonly used preservative and chemical microbial agent that was subjected to risk management activities in 2020 (Government of Canada 2020); diphenhydramine (median = 547 ng/g), an antihistamine that is available without a prescription (Wolfson et al. 2022); doxycycline (median = 690 ng/g), a broad-spectrum antibiotic of the tetracycline class (Saatchi et al. 2021); clotrimazole (median = 828 ng/g), an antifungal medication that is commonly available without a prescription (Crowley and Gallagher 2014); and amitriptyline (median = 359 ng/g), an antidepressant (Luo et al. 2020) that is also used off-label to treat chronic pain, anxiety, and insomnia (Urits et al. 2019; Bakker et al. 2023). These concentrations were within the range observed in biosolids previously (Lajeunesse et al. 2012; Guerra et al. 2014; Verlicchi and Zambello 2015; Tran et al. 2018; Martin and Hart 2023).

**Fig. 5:**
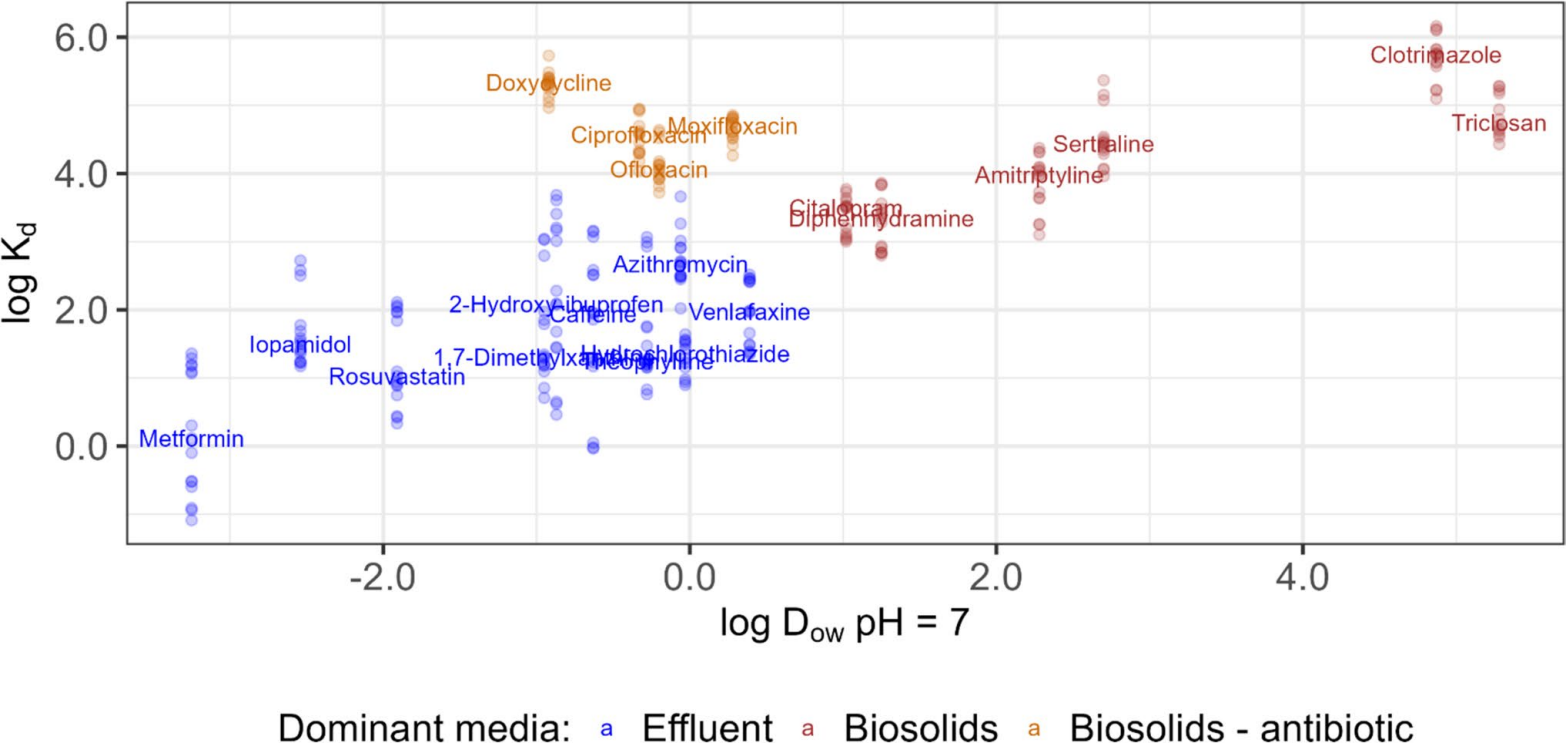
Calculated log solid–liquid distribution coefficients (*K*_d_) as a function of log *D*_ow_ (pH = 7) for selected PPCPs present at elevated concentrations in either effluent (blue) or biosolids (all substances except for antibiotics, brown; antibiotics, orange). Data collected in 2022 were included in this figure. The chemical name indicates the median calculated log *K*_d_ values and the dots represent individual data points

PPCPs with elevated concentrations in biosolids (Fig. 4) differed from those elevated in influent and effluent (Figs. 1 and 3). Log *K*_d_ values, reported in Table S1-23 and presented in Fig. 5 and Fig. S2.2, provide information on the potential for each PPCP to be released into the environment from biosolids versus effluent. To determine if hydrophobicity is a factor influencing *K*_d_ values, log *K*_d_ values from 2022 were plotted as a function of either log *D*_ow_ at pH = 7 or log *K*_ow_, for ionic and non-ionic PPCPs, respectively. Log *D*_ow_ is the pH-dependent log octanol–water partition coefficient (log *K*_ow_) (Wells 2006; Kah and Brown 2008) and is used instead of log *K*_ow_ as a measure of hydrophobicity for ionic substances. Log *D*_ow_ accounts for the fact that polarity, and hence hydrophobicity, changes with pH depending on whether the substance is in an ionized or neutral form at the specified pH (Wells 2006; Kah and Brown 2008). A pH of 7 was assumed; the median pH in effluent samples collected in 2022 ranged from 7.2 to 8.1, with a median of 7.46. Figure 5 shows log *K*_d_ as a function of log *D*_ow_ at pH = 7 for the top 10 dominant PPCPs in effluent and biosolids. The dominant PPCPs in effluent and biosolids were ionic, and hence, log *D*_ow_ was used as a measure of hydrophobicity in Fig. 5. Figure S2.2 shows the relationship between median log *K*_d_ values as a function of log *D*_ow_ at pH = 7 (ionic PPCPs) or log *K*_ow_ (non-ionic PPCPs) for PPCPs detected in greater than or equal to 50% of samples in either effluent or biosolids. For the most part, log *K*_d_ increased as a function of log *D*_ow_ or log *K*_ow_. PPCPs dominant in effluent that would be released primarily to the aquatic environment had log *D*_ow_ or log *K*_ow_ values less than 0.4 and median log *K*_d_ values ranging from 0.12 (metformin) to 2.7 (azithromycin) (Table S1-23 and Fig. 5). PPCPs dominant in biosolids that would be released primarily to the terrestrial environment had log *D*_ow_ or log *K*_ow_ values greater than 1.0 and median log *K*_d_ values ranging from 3.3 (diphenhydramine) to 5.7 (clotrimazole) (Table S1-23 and Fig. 5). Exceptions occurred for select antibiotics in the fluoroquinolone and tetracyclines classes which had elevated log *K*_d_s, with median values ranging from 3.5 (minocycline) to 5.3 (doxycycline) and therefore would be released to the environment primarily via biosolids but had relatively low log *D*_ow_ or log *K*_ow_ less than 0.3 (Table S1-23, Fig. 5, and Fig. S2.2). Overall, these results indicate that chemical hydrophobicity influenced whether these PPCP were released to the environment via effluent or biosolids with the exception of fluoroquinolone and tetracyclines antibiotics, where sorption was likely governed by factors such as electrostatic forces, which has been found in other studies (Guerra et al. 2014; Berthod et al. 2017; Son et al. 2022). Several previous studies have measured *K*_d_ values in full-scale WWTPs, where values varied considerably (Sathyamoorthy and Ramsburg 2013; Tran et al. 2018). Our calculated *K*_d_ values were within the range of values previously calculated for PPCPs in Canadian WWTPs (Table S1.23) (Guerra et al. 2014) and in WWTPs located in other countries (Sathyamoorthy and Ramsburg 2013; Tran et al. 2018).

### Removal of PPCPs

**Fig. 6:**
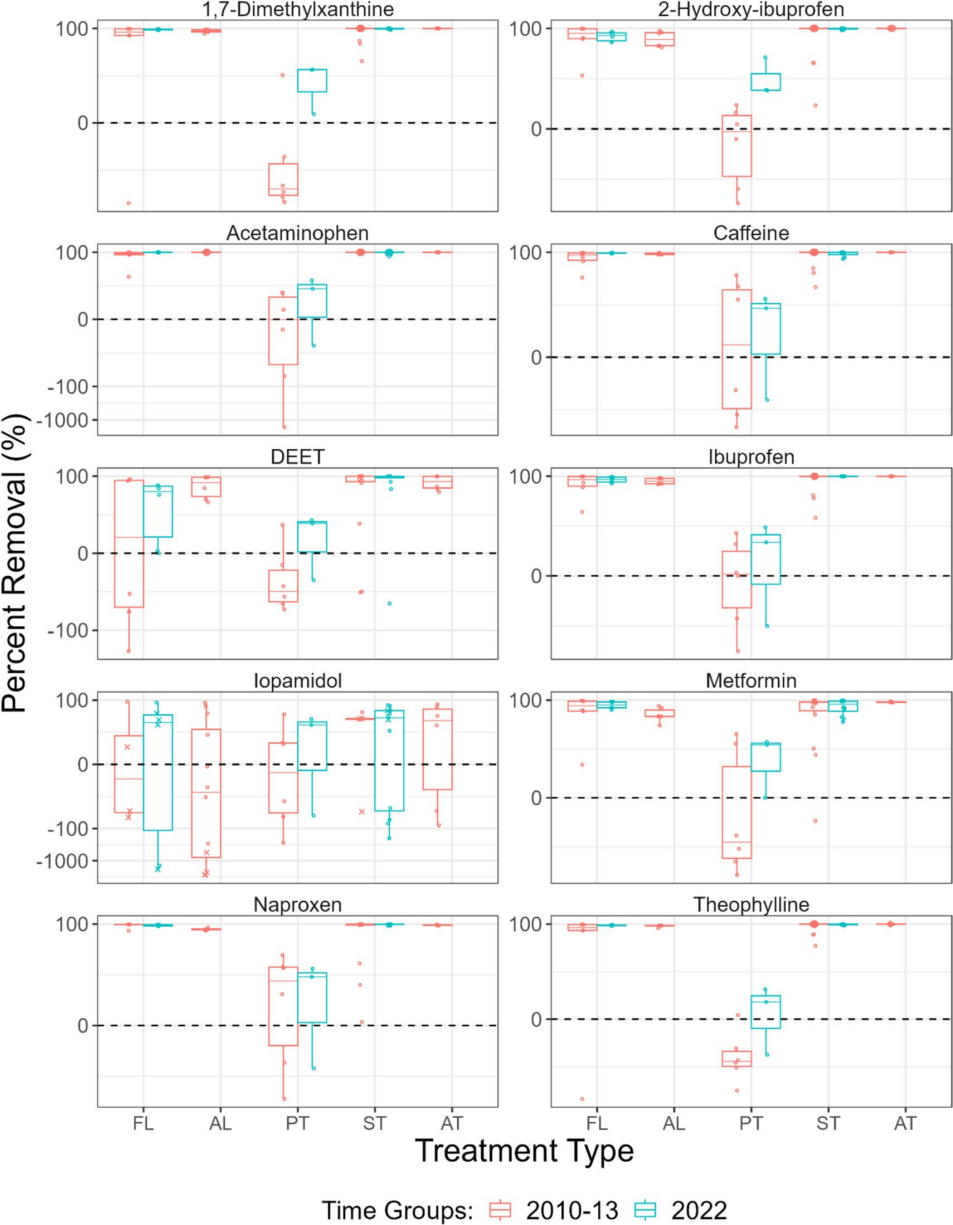
Percent removal of ten representative PPCPs by treatment type in 2010–2013 and 2022. Data are presented for facultative lagoons (FL), aerated lagoons (AL), primary treatment plants (PT), secondary treatment plants (ST), and advanced treatment plants (AT). The line within the boxes shows median removal, the boxes show 25th and 75th percentiles, and the whiskers below and above the boxes show 10th and 90th percentiles. The dots indicate individual data points. Circles show removals calculated using detects. Diamonds show cases where PPCPs were detected in influent but not in effluent, the detection limit concentration in effluent was used to estimate a removal value, and the resulting calculation represents the lower bound of percent removal, with the upper bound being 100%. The symbol ( ×) shows cases where PPCPs were detected in effluent but not in influent, the detection limit concentration in influent was used to estimate a removal value, and the resulting calculated removal represents the upper bound of percent removal

Percent removal of PPCPs during wastewater treatment is shown in Table S1-24. For samples collected in 2022, percent removal for representative PPCPs dominant in influent and effluent are shown in Fig. 6, and median percent removal as a function of median log *K*_d_ is plotted in Fig. S2.3 for primary and secondary WWTPs. For many PPCPs, higher removal was observed at plants that use biological treatment (lagoons, secondary and advanced treatment) compared to primary physico/chemical treatment (Fig. 6 and Fig. S2.3). This has been observed in past studies (Guerra et al. 2014, 2019; Yang et al. 2017; Di Marcantonio et al. 2020). Primary treatment processes tended to be inefficient for removing PPCPs because of their use of sedimentation to remove solid waste during wastewater treatment. Previous studies have found that sorption was a major mechanism for the removal of PPCPs in primary treatment plants (Suárez et al. 2008; Yang et al. 2017). There was no significant relationship between percent removal and log *K*_d_ for PPCPs at primary treatment plants (*p* = 0.082, Kendall’s Tau = 0.14) (Fig S2.3). However, PPCPs with median log *K*_d_ values greater than 4.2 tended to have higher removals (greater than 50%) compared to PPCPs with lower solid sorption. The short hydraulic retention time of primary treatment plants (Table S1-1) also led to reduced removals compared to plants that used biological treatment (Guerra et al. 2014; Yang et al. 2017). The greater PPCP removal in lagoons, secondary, and advanced treatment plants compared to primary WWTPs was likely a result of PPCP biotransformation as well as greater hydraulic retention time (Table S1.1) (Suárez et al. 2008; Guerra et al. 2014; Yang et al. 2017). In addition, the nitrification conditions observed in some of the lagoons, secondary and advanced treatment types (Table S1.1) were correlated with improved removal efficiency for some PPCPs (Guerra et al. 2014; Yang et al. 2017). There was a significant relationship between percent removal and log *K*_d_ for PPCPs in secondary treatment plants (*p* = 0.023) but the relationship was very weak (Kendall’s Tau = 0.17) (Fig S2.3). This indicates that removal via sorption remained a mechanism for the removal of PPCPs at plants that use biological treatment (Suárez et al. 2008; Guerra et al. 2014; Yang et al. 2017).

In some cases, negative removals were observed. Since WWTPs do not use or generate PPCPs, negative removals could be due to one or a combination of the following factors: First, the influent and effluent samples were filtered by the laboratory prior to analysis. Although this was a necessary step in the analysis of PPCPs at WWTPs (Bade et al. 2019; Gewurtz et al. 2022; Liu et al. 2023), filtering removes a greater portion of the analytes from influent compared to effluent (Deo and Halden 2010; Guerra et al. 2019; Wu et al. 2021). This process was more important for chemicals that partition or sorb strongly to solids (i.e., substances with log *D*_ow_ greater than 1.0 and fluoroquinolone and tetracycline antibiotics) compared to other PPCPs (Fig. 5 and Fig. S2.2). Second, many PPCPs are excreted by humans in conjugated forms that are not detected by analytical techniques aimed at free analytes. Conjugation can be reversed by naturally occurring enzymes that are found in WWTPs, which could lead to apparent negative removals (Comber et al. 2019; Gewurtz et al. 2022; Kumar et al. 2022). Third, PPCPs could be formed from unmeasured precursor chemicals during wastewater treatment even though this transformation does not increase the total mass of the PPCPs in the WWTPs (Liu et al. 2012, 2015; Kumar et al. 2022).

### Time trends

Changes in PPCP concentrations between 2010–2013 and 2022 were assessed to evaluate time trends. Figure S2.4 and Table S1.25 show the time trends of PPCPs and their significance by category in influent, effluent, and biosolids. Here, we highlight examples of time trends. Detailed comments on temporal changes are provided in Table S1.25.

#### PPCPs that did not change between 2010–2013 and 2022

**Fig. 7:**
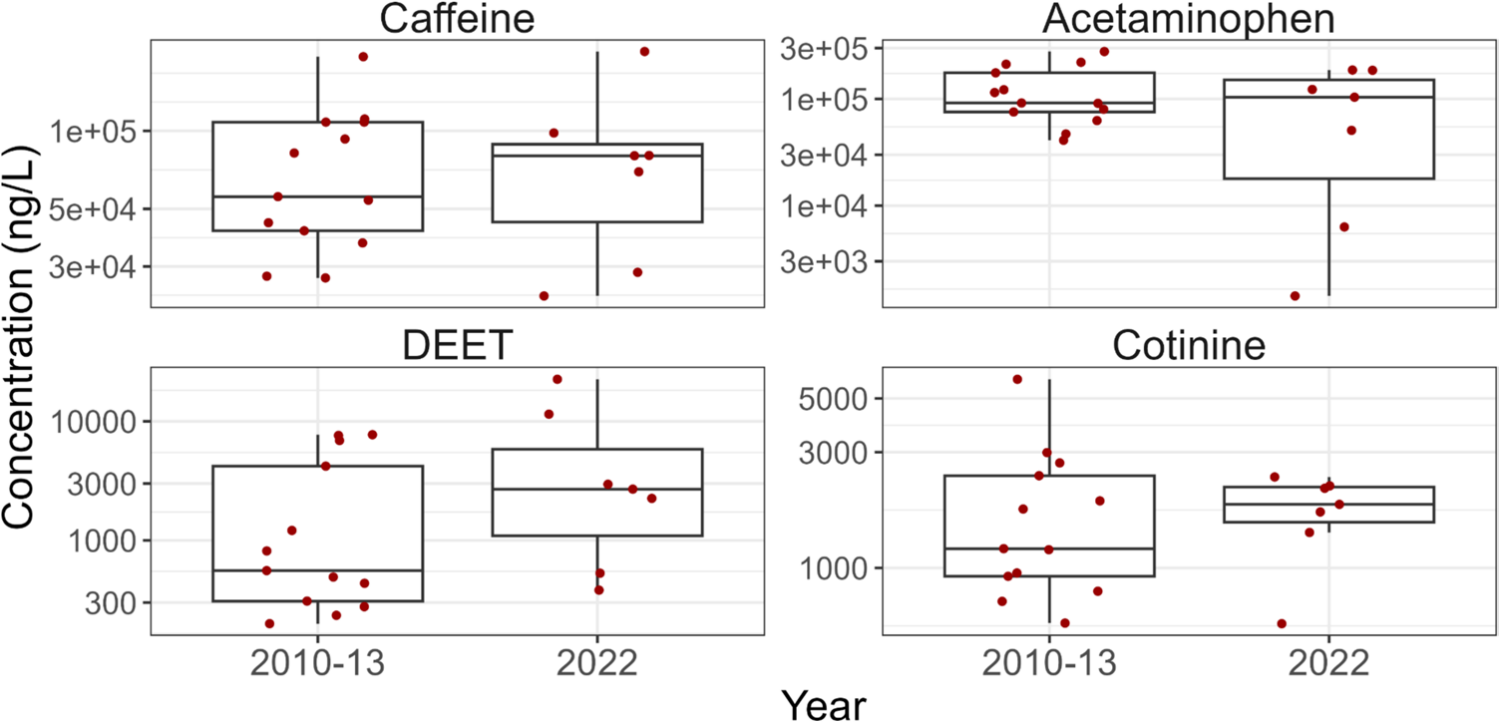
Comparison of the concentrations of four representative PPCPs in influent between 2010–2013 and 2022 that did not change significantly between the two time periods. The data points used in this figure represent the median PPCP concentration measured on three consecutive days at each WWTP and sampling event. The line within the boxes shows median concentrations, the boxes show 25th and 75th percentiles, and the whiskers below and above the boxes show 10th and 90th percentiles. Dots show individual data points. Red dots show detected concentrations and grey dots show concentrations below detection and plotted at the detection limit. Note the log scale

The concentrations of many PPCPs did not change significantly (*p* > 0.05) in wastewater and biosolids between 2010–2013 and 2022 (Fig. 7, Fig. S2.4, and Table S1.25). For example, caffeine and its metabolite (1,7 − dimethylxanthine) did not change significantly in influent, effluent, and biosolids (*p* > 0.05) between the two time periods. Caffeine is present in popular food and beverages and is one of the most widely consumed stimulants in the world (Quadra et al. 2020; Korekar et al. 2020). In comparison to our results, global caffeine intake may have slightly increased due to the introduction of new caffeinated beverages and/or population growth (Quadra et al. 2020; Korekar et al. 2020).

Concentrations of over-the-counter pharmaceuticals including analgesic/anti-inflammatories (i.e., acetaminophen, ibuprofen and its metabolite 2-hydroxy-ibuprofen, and naproxen), as well as the antihistamine, diphenhydramine, also did not change significantly (*p* > 0.05) with time in wastewater matrices. The lack of time change for these chemicals was likely because they are easily accessible and popular for treating common ailments.

Cotinine is a metabolite of nicotine. There has been a reduction in smoking (Reid et al. 2022) and cigarette sales (Government of Canada 2021) in Canada since 2010. Our data contradicted this pattern with no changes in cotinine concentrations observed between 2010–2013 and 2022. This could be due to an increasing prevalence of non-tobacco nicotine products (vaping, nicotine replacement therapies, etc.), as found in a recent Australian study (Wang et al. 2024).

There was some indication that median concentrations of DEET were increasing in wastewater and biosolids (Fig. 7 and Fig. S2.4), although the increasing concentration trends were not significant (*p* > 0.05, Table S1.25). Health Canada recommends use of personal insect repellent for protection against insect bites and potentially serious diseases such as Lyme disease, and the incidence of Lyme disease has increased since 2009 (Government of Canada 2024).

#### PPCPs that increased between 2010–2013 and 2022

**Fig. 8:**
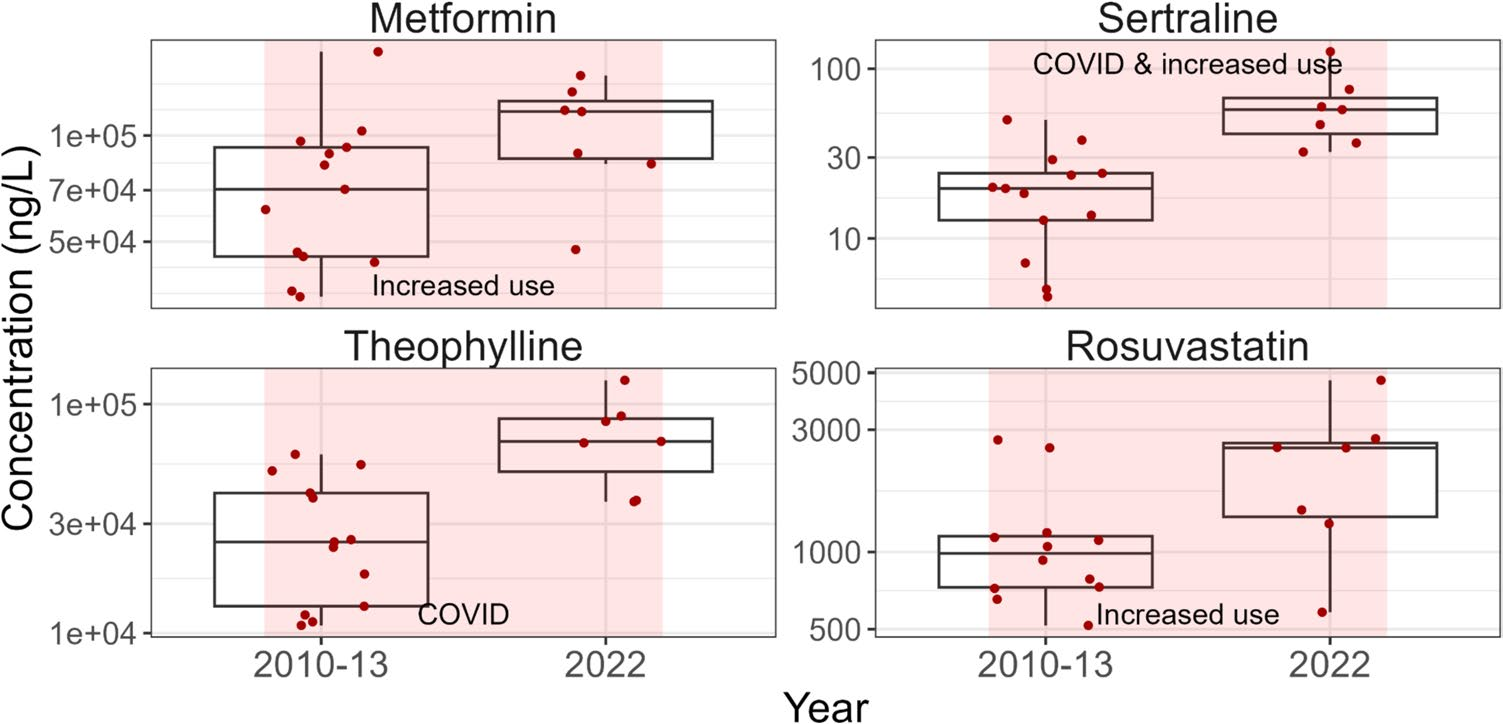
Comparison of the concentrations of four representative PPCPs in influent between 2010–2013 and 2022 which increased significantly (*p* < 0.05) between the two time periods. The data points used in this figure represent the median PPCP concentration measured on three consecutive days at each WWTP and sampling event. The line within the boxes shows median concentrations, the boxes show 25th and 75th percentiles, and the whiskers below and above the boxes show 10th and 90th percentiles. Dots show individual data points. Red dots show detected concentrations and grey dots show concentrations below detection and plotted at the detection limit. Shaded rectangles indicate a significant (*p* < 0.05) increase (pink) in PPCP concentrations between the two time periods. Potential factors (if any) that influence the time changes are indicated in black text with additional comments and details identified in Table S1.25. Note the log scale

PPCP concentrations that increased significantly (*p* < 0.05) between 2010–2013 and 2022 are presented in Fig. 8, Fig. S2.4, and Table S1.25. Metformin concentrations increased significantly (*p* < 0.05) in Canadian influent. This corresponded to its use patterns in Canada (Secrest et al. 2020). The prevalence of the rate of diagnosed diabetes is increasing in Canada (Government of Canada 2022). In 2008, the Canadian Diabetes Association (CDA) recommended metformin as the initiating treatment for patients newly diagnosed with type 2 diabetes due to the lower incidence of side effects and its relatively cheap cost compared to other medications (Diabetes Canada Clinical Practice Guidelines Expert Committee 2018). A population-based analysis of antidiabetic medications in four Canadian provinces found that by the early 2000s, metformin was the most widely prescribed antidiabetic medication (Secrest et al. 2020). Another study found that after the CDA guideline change in 2008, there was an increased incidence of metformin use in newly diagnosed patients in Quebec, Canada (Wang et al. 2013). In addition to treating type 2 diabetes, metformin is used to treat polycystic ovary syndrome and nonalcoholic fatty liver disease (Petrie 2024). It is also used for weight management and promotion of healthy ageing, and it is being tested for cancer treatment (Petrie 2024). These applications could have also increased the administration of metformin in Canada. Metformin did not change over the two time periods in wastewater effluent and biosolids (Fig. S2.4 and Table S1.25). The reason for the differences in time trends of metformin in effluent and biosolids compared to influent could be variability in data and slight changes to removal over time. As shown in Fig. 6 and Table S1.24, although the removal of metformin was highly variable, there were more instances of low or negative removals in 2010–2013 compared to 2022.

Sertraline concentrations increased significantly (*p* < 0.001) in influent and biosolids between 2010–2013 and 2022 and increased (albeit not-significantly, *p* > 0.05) in effluent (Fig. 8, Fig. S2.4, and Table S1.25). This increase of sertraline in wastewater matrices suggested increased use in Canada. In 2004, Health Canada advised Canadians that SSRIs, including sertraline, would carry stronger warnings to indicate that patients may experience behavioral and/or emotional changes that put them at increased risk of self-harm or harm to others (Government of Canada 2004). Although this warning was associated with an immediate drop in SSRI prescriptions in Canadian children and adolescents, this decrease was not sustained, and after 5 years, SSRI prescription rates increased (Morkem et al. 2017). The Canadian Pediatric Society indicated sertraline as a second line of treatment for depression, and there has been a shift towards prescribing sertraline to children and adolescents since 2009 (Morkem et al. 2017). In the US, sertraline was among the most prescribed antidepressants to adults with major depression between 1996 and 2015 and the number of prescriptions increased during this time period (Luo et al. 2020). As mentioned above, sertraline was shown to exhibit antiviral activity against SARS-CoV-2 and to alleviate symptoms associated with infection (Chen et al. 2022). Therefore, it is possible that concentration increases of sertraline in Canadian wastewater between 2010–2013 and 2022 were associated with the COVID-19 pandemic. Additional data between 2013 and 2020 may strengthen this hypothesis.

Theophylline concentrations increased significantly (*p* < 0.001) in Canadian wastewater influent, but not in effluent (high degree of removal) or biosolids (little partitioning to solids). As previously mentioned, theophylline is a respiratory medication (Montaño et al. 2022; Boylan et al. 2023) that was found to be an effective treatment for the symptoms of the SARS-CoV-2 virus (Montaño et al. 2022). Therefore, increased concentrations of this substance in Canadian wastewater influent between 2010–2013 and 2022 may be related to the COVID-19 pandemic. As with sertraline, additional data between 2013 and 2020 may strengthen this hypothesis.

Rosuvastatin concentrations increased significantly (*p* < 0.05) in Canadian wastewater influent between 2010–2013 and 2022 (Fig. 8). Although concentrations of rosuvastatin in effluent and biosolids showed some indication of increasing trends between 2010–2013 and 2022 with a greater detection frequency in the later time period (Fig. S2.4), these increases were not significant (*p* > 0.05). Statins such as rosuvastatin are one of the most frequently prescribed classes of pharmaceuticals in Canada (Hennessy et al. 2016). They were recommended by the Canadian Cardiovascular Society for high-risk patients and some intermediate-risk patients for the management of dyslipidemia for the prevention of cardiovascular disease (Pearson et al. 2021). In 2013, Health Canada advised Canadians about a labeling update for statins because of a risk of increased blood sugar levels and a small increased risk of diabetes among patients already at risk for the disease (Government of Canada 2013). Health Canada continues to believe that the overall cardiovascular benefits of statin drugs in reducing blood cholesterol outweigh their risks (Government of Canada 2013). This labeling update did not result in a decrease in rosuvastatin concentrations in Canadian wastewater between 2010–2013 and 2022.

#### PPCPs that decreased between 2010–2013 and 2022

**Fig. 9:**
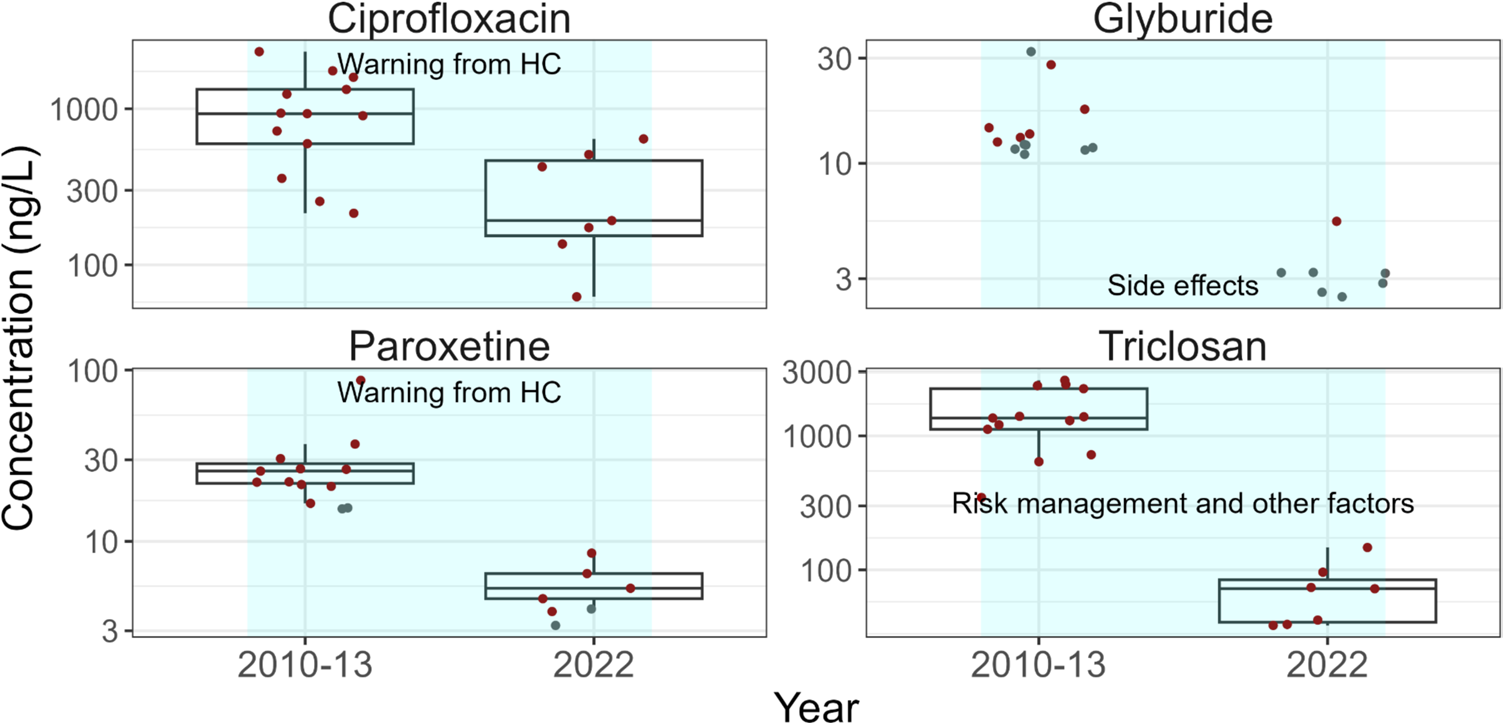
Comparison of the concentrations of four representative PPCPs in influent between 2010–2013 and 2022 which decreased significantly (*p* < 0.05) between the two time periods. The data points used in this figure represent the median PPCP concentration measured on three consecutive days at each WWTP and sampling event. The line within the boxes shows median concentrations, the boxes show 25th and 75th percentiles, and the whiskers below and above the boxes show 10th and 90th percentiles. Dots show individual data points. Red dots show detected concentrations and grey dots show concentrations below detection and plotted at the detection limit. Shaded rectangles indicate a significant (*p* < 0.05) decrease (blue) in PPCP concentrations between the two time periods. Potential factors (if any) that influence the time changes are indicated in black text with additional comments and details identified in Table S1.25. Note the log scale

The concentrations of many of the antibiotics included in this study decreased significantly (*p* < 0.05) in Canadian wastewater matrices between 2010–2013 and 2022 with no increases observed for any of the antibiotics (Table S1.25). These decreases could be due to increased awareness of antimicrobial resistance and efforts to reduce antibiotic use (Rudnick et al. 2020). One specific antibiotic that decreased significantly (*p* < 0.05) in influent, effluent, and biosolids between 2010–2013 and 2022 (Fig. 9, Fig. S2.4, and Table S1.25) was ciprofloxacin. This corresponded to a study of antibiotic use in adult inpatients at Canadian hospitals where ciprofloxacin use decreased between 2009 and 2016 (Rudnick et al. 2020). The decrease in use may have been associated with the 2008 Health Canada warning that fluoroquinolone antibiotics such as ciprofloxacin could lead to tendon ruptures (Government of Canada 2017).

Concentrations of glyburide, a sulfonylureas antidiabetic drug, decreased significantly in influent and effluent (*p* < 0.01) between 2010–2013 and 2022 (Fig. 9, Fig. S2.4, and Table S1.25). In biosolids, concentrations of glyburide showed evidence of a decrease but the trend was not significant (*p* > 0.05) (Fig. S2.4). Studies of glyburide use in Canada corresponded to our trends. As of 2008, the CDA no longer recommends sulfonylureas such as glyburide as the initiating treatment for type 2 diabetes due to side effects including weight gain and hypoglycemia (Wang et al. 2013; Diabetes Canada Clinical Practice Guidelines Expert Committee 2018; Secrest et al. 2020). A population-based analysis of antidiabetic medications in four Canadian provinces found that in the 1990s, sulfonylureas were the most prescribed antidiabetic medication of those studied (Secrest et al. 2020). However, in the early 2000s, prescription rates of sulfonylureas decreased in favor of metformin (Secrest et al. 2020). Another study found that after the new CDA guideline change in 2008, there was a decreased incidence of use of sulfonylureas in newly diagnosed patients in Quebec, Canada (Wang et al. 2013).

Concentrations of the SSRI paroxetine decreased significantly (*p* < 0.01) between 2010–2013 and 2022 in wastewater influent and effluent and showed a decreasing trend in biosolids, though it was not significant. As mentioned above, in 2004, Health Canada advised Canadians that SSRIs, such as paroxetine, would carry stronger warnings to indicate that patients taking these drugs may experience behavioral and/or emotional changes that could put them at greater risk of self-harm or harm to other people. Paroxetine was the leading SSRI prescribed to Canadian children and adolescents before the 2004 Health Canada advisory (Government of Canada 2004) but its use decreased since then.

Triclosan concentrations decreased significantly (*p* < 0.05) between 2010–2013 and 2022 in wastewater influent and effluent as well as biosolids. As discussed above, triclosan had the third highest median concentration in biosolids of all the PPCPs analyzed in this study but was subjected to risk management activities in 2020 (Government of Canada 2020).

## Conclusions and implications

In an evaluation of 135 PPCPs at seven representative Canadian WWTPs in 2022, this study found relatively elevated concentrations of metformin, acetaminophen, caffeine, and theophylline in influent and/or effluent, and ciprofloxacin, sertraline, triclosan, and diphenhydramine in biosolids. These elevated concentrations likely reflected the use of these PPCPs in Canada. Elevated PPCPs in influent/effluent differed from those in biosolids. Hydrophobicity of the PPCPs was a key factor influencing whether a PPCP was dominant in effluent or biosolids; PPCPs with log *D*_ow_ or log *K*_ow_ values less than 0.4 tended to be present at higher concentrations in effluent and thus would be primarily released to the aqueous environment whereas PPCPs with log *D*_ow_ or log *K*_ow_ greater than 1.0 were primarily found in biosolids and thus the primary release pathway would be to the terrestrial environment. Exceptions occurred for select antibiotics, e.g., fluoroquinolone and tetracyclines, which were dominant in biosolids despite their relatively low hydrophobicity as their sorption was likely governed by electrostatic forces. As observed in previous studies (Guerra et al. 2014, 2019), higher removal from influent to effluent was observed at plants that use biological treatment (lagoons, secondary and advanced treatment) compared to primary treatment.

The time trends of PPCPs in wastewater matrices between 2010–2013 and 2022 varied and may have been influenced by factors such as risk management measures, warnings (e.g., antibiotics), development of new pharmaceuticals (e.g., antidiabetics and antidepressants), and the COVID-19 pandemic (e.g., sertraline and theophylline). In Canada, there is limited information on PPCP use in the community and release into the environment. Our wastewater data reflected the limited information available and therefore continued periodic monitoring of PPCPs in wastewater matrices is recommended to fill data gaps.

## Supplementary Information

Below is the link to the electronic supplementary material.

Supplementary file1 (XLSX 440 KB)

Supplementary file2 (DOCX 5.35 MB)

## Data Availability

The raw data for this report may be accessed at the Government of Canada (2025).
