# Supplementary material for: Pharmaceuticals and personal care products in Canadian municipal wastewater and biosolids: occurrence, fate, and time trends 2010–2013 to 2022
Source: Environ Sci Pollut Res Int. 2025 Feb 3;32(9):5022–39. doi: 10.1007/s11356-025-36007-0 (PMC11868229; doi:10.1007/s11356-025-36007-0)
Supplement: Supplementary file 2 — Supplementary file2 (DOCX 5.35 MB) [file 11356_2025_36007_MOESM2_ESM.docx]

**Supplementary Information 2**

**Pharmaceuticals and personal care products in Canadian municipal wastewater and biosolids: occurrence, fate, and time trends 2010–2013 to 2022**

**Sarah B. Gewurtz*, Alexandra S. Auyeung, Steven Teslic, Shirley Anne Smyth**

^a^ Science and Technology Branch, Environment and Climate Change Canada, Burlington, Ontario L7S 1A1, Canada

*Corresponding author at: Science and Technology Branch, Environment and Climate Change Canada, Burlington, Ontario L7S 1A1, Canada.

*E-mail address*: sarah.gewurtz@ec.gc.ca

# Table of Contents

[Table of Contents 2](#_Toc185239436)

[Table of Figures 3](#_Toc185239437)

[Analytical methods 4](#_Toc185239438)

[Chemical and instrumental analysis for PPCPs 4](#_Toc185239439)

[Conventional wastewater analysis 6](#_Toc185239440)

[Quality assurance and quality control 6](#_Toc185239441)

[Results for conventional parameters 9](#_Toc185239442)

[Figures 10](#_Toc185239443)

[References 39](#_Toc185239444)

# Table of Figures

[Fig. S2.1 a-g. Median concentrations of PPCPs in 2022 wastewater influent as a function of population served. 10](#_Toc185241111)

[Fig. S2.2. Median calculated log solid-liquid distribution coefficients (K_d_) as a function of either log D_ow_ (pH = 7) for ionic PPCPs or log K_ow_ for non-ionic PPCPs. Data collected in 2022 were included in this figure. 17](#_Toc185241112)

[Fig. S2.3. Median percent removal as a function of median calculated log solid-liquid distribution coefficients (K_d_) for primary and secondary treatment facilities. Data collected in 2022 were included in this figure. 18](#_Toc185241113)

[Fig. S2.4 a-g. Comparison of PPCP concentrations in influent, effluent, and biosolids between 2010-13 and 2022. 19](#_Toc185241114)

# Analytical methods

## Chemical and instrumental analysis for PPCPs

As described in the main text, 135 pharmaceuticals and personal care products (PPCPs) were analyzed by SGS AXYS Analytical Services Limited (SGS AXYS) according to SGS AXYS in-house method MLA-075 (Table S1.2 of Supplementary Information (SI) 1). This method is based on USEPA method 1694 (USEPA 2007). SGS AXYS is accredited by the Canadian Association for Laboratory Accreditation Inc. (CALA). SGS AXYS has participated in numerous round robins and intercalibration studies where its proficiency in PPCP analysis was demonstrated.

Upon arrival at the laboratory, the samples were stored frozen under dark conditions at ‑20 ºC until analysis. The wastewater influent and effluent samples were filtered after thawing and the dissolved phase of these samples were analyzed. Both aqueous and biosolids samples were extracted at two different pH conditions (Table S1.2). Aqueous samples were adjusted to a pH of 2 or 10 for the acidic and basic extraction conditions, respectively. Labeled extraction internal (surrogate) standards were spiked into the samples and mixed. At this stage, the aqueous samples were ready for the weak anion exchange (WAX) solid phase extraction (SPE) procedures described below.

Biosolids samples were adjusted to a pH of 2 or 10 for the acidic and basic extraction conditions, respectively. The biosolids samples were then spiked with labeled extraction internal (surrogate) standards. The biosolids samples were sonicated with acetonitrile and the extract concentrated by rotary evaporation. At this stage, the biosolids samples were ready for the WAX SPE clean-up procedures described below.

After the above preparations, aqueous and biosolids samples were cleaned up by SPE (Waters Oasis HLB). The acidic extracts were loaded onto the SPE cartridge, washed with ultra pure water, and eluted with methanol and acetone:methanol (1:1). The basic extracts were loaded onto the SPE cartridge and then eluted with methanol and formic acid. Recovery standards were added to cleaned-up samples. Extracts that were cloudy or contained floating particulates were filtered prior to instrumental analysis.

The samples were analyzed using liquid chromatography-electrospray ionization tandem mass spectrometry (LC/ESI-MS/MS) run in positive or negative ionization modes (Table S1.2). The instrument used was a Waters 2690 or Waters 2795 HPLC coupled to a Micromass Quattro Ultima MS/MS. The HPLC column implemented was a Waters Xterra C18MS, 10.0 cm, 2.1 mm i.d., 3.5 µm particle size. Initial calibration was performed by the analysis of seven or more calibration solutions. A single point (mid-level) calibration standard was analyzed to verify the initial calibration after every 12 hours or every 20 samples, whichever occurred first. Target compounds were quantified against the isotopically labelled surrogate standards detailed in Table S1.3. Given that isotope dilution was used, the measured concentrations in each sample were inherently corrected for recovery of the surrogate standards, with the limitation that exact surrogates were not available for every analyte. In those cases, one of the other labeled surrogates (chemically similar) was used to quantify the analyte by internal standard quantification. Sample specific detection limits were calculated using three times the signal of the noise in the target channel converted to an equivalent sample concentration. The reporting limits (Table S1.4) were the greater of the sample specific detection limits or the concentration equivalent to the lowest calibration standard.

## Conventional wastewater analysis

Influent, effluent, and biosolids samples were analyzed for conventional wastewater parameters by the National Laboratory for Environmental Testing (NLET) of Environment and Climate Change Canada (ECCC). Influent and effluent samples were analyzed for pH, alkalinity, total suspended solids (TSS), chemical oxygen demand (COD), total organic carbon (TOC), total Kjeldahl (organic) nitrogen (TKN), ammonia, and nitrate. Solids samples were analyzed for total and volatile solids (TS and VS). Conventional analyses were conducted as per standard methods for the examination of water and wastewater (American Public Health Association 2012).

## Quality assurance and quality control

Quality assurance/quality control (QA/QC) elements for the PPCP analyses included spiked reference samples, surrogate recoveries, laboratory duplicates, laboratory blanks, equipment blanks, and evaluation of variability between samples collected on three consecutive days. QA/QC data are presented separately for the following periods: 2010-2013 and 2022. There was a version change between these two time periods that included minor changes to surrogates used to quantify analytes (Table S1.3).

Five percent of samples within each batch were spiked reference samples (i.e., one spiked reference sample for every 20 wastewater or biosolids samples or fewer). Spiked reference samples were used to determine the effectiveness of the analytical procedure. The spiked reference samples were prepared by spiking an aliquot of a native standard solution into a clean matrix and then extracting the spiked solution in a manner analogous to a real sample. The clean matrix for the aqueous samples consisted of ultrapure water. The clean matrix for the solid samples consisted of cleaned biosolid. Spike recoveries are presented in Tables S1.5 and S1.6. Spiked matrix recoveries were within the acceptable range limits outlined in the analytical method except as noted in Tables S1.5 and S1.6.

Isotopically labeled surrogate standards were spiked into every field and lab sample as part of the isotope-dilution quantification process. Table S1.3 shows the surrogates used to quantify each of the target analytes. The recoveries of these surrogates were monitored to determine the effectiveness of the analytical system. Surrogate recoveries are presented in Tables S1.7 and S1.8. Given that isotope dilution was used, the measured concentration in each sample was inherently corrected using recovery of the surrogate standards, with the limitation that exact surrogates were not available for every analyte (Table S1.3).

Aqueous and biosolids were analyzed in duplicate in each time group to provide an indication of the precision of the extraction and analytical method. The closeness of duplicate analyses was determined using the percent difference between values ((higher value–lower value)/average value)*100). The ranges of the percent difference of duplicate samples are presented in Tables S1.9 and S1.10. Most percent difference values for duplicates are within the SGS AXYS guideline of 40% and are within the range typical of contaminant studies. Non-detect concentrations were not included in the percent difference calculations.

Five percent of samples within each batch were laboratory blanks (i.e., one blank for every 20 wastewater or biosolids samples or fewer). The blank was prepared by spiking an aliquot of the surrogate standard solution into a clean matrix consisting of ultrapure water. Laboratory blank results are presented in Tables S1.11 and S1.12. PPCP concentrations in laboratory blanks were mostly below the detection limit with exceptions highlighted in Tables S1.11 and S1.12.

Aqueous and solid equipment blanks were prepared and analyzed as a measure of potential background contamination to samples because of our cleaning procedure and sampling equipment. The aqueous equipment blanks consisted of HPLC-grade water (Caledon Laboratories, Georgetown, Canada) run through sampling equipment and containers. The solid equipment blanks consisted of silica sand (Fisher Scientific, Ottawa, Canada) that was poured into the cleaned biosolids bucket and mixed with the metal spoon and gloves in the same manner as used for sampling. Equipment blank results are presented in Tables S1.13 and S1.14. PPCP concentrations in equipment blanks were mostly below the detection limit with exceptions highlighted in Tables S1.13 and S1.14.

Some concentrations of PPCPs detected in laboratory and equipment blanks were on the lower end of the range of concentrations detected in influent, effluent, and biosolids samples (Tables S1.15 and S1.16). PPCPs with concentrations in equipment and/or laboratory blanks that exceeded measured concentrations in influent, effluent, and/or biosolids are presented in Tables S1.15 and S1.16. Given that concentrations of a few PPCPs in laboratory and equipment blanks only exceeded the lowest concentrations measured in some samples, background contamination (when present) did not affect the findings of this study and blank subtraction was not performed.

As described in the main text, samples were collected for three consecutive days to provide information about the variability in the sampling and analytical systems. The relative standard deviation (RSD) was used to assess the variability in results: RSD = standard deviation/mean expressed as a percentage. RSD values are presented in Tables S1.17-S1.19. The degree of variability in concentrations reflects the complexity and variability of the sample matrix and the variability in the extraction and analytical system. The ranges of variability of PPCPs in influent, effluent, and biosolids are typical for trace contaminants in these matrices.

# Results for conventional parameters

Levels of conventional parameters were monitored to provide context for the concentrations and removals of PPCPs in wastewater treatment processes. Wastewater treatment plants (WWTPs) were receiving low, medium, and high strength wastewater and achieving typical removals of COD and TSS for each treatment type, indicating that the WWTPs were operating normally during the sampling periods (Metcalf & Eddy Inc 2003). Data for conventional parameters are available at Government of Canada (2024).

# Figures

Fig. S2.1 a-g. Median concentrations of PPCPs in 2022 wastewater influent as a function of population served.

WWTP codes indicate median influent concentrations; plain red font indicates that median concentrations were above the detection limit whereas italicized red font indicates that median concentrations were below the detection limit. Circles indicate individual data points; red dots = detected concentrations, grey dots = concentrations below detection and plotted at the detection limit. The p-values indicate the significance of the Kendall’s correlation coefficient, with p-values in red and black font indicating significant (p < 0.05) and non-significant (p > 0.05) relationships, respectively. PPCPs are shown if detected in greater than or equal to 30% of influent samples. Note the log scale.

**Fig. S2.1 a** (Analgesic/anti-inflammatories)

**
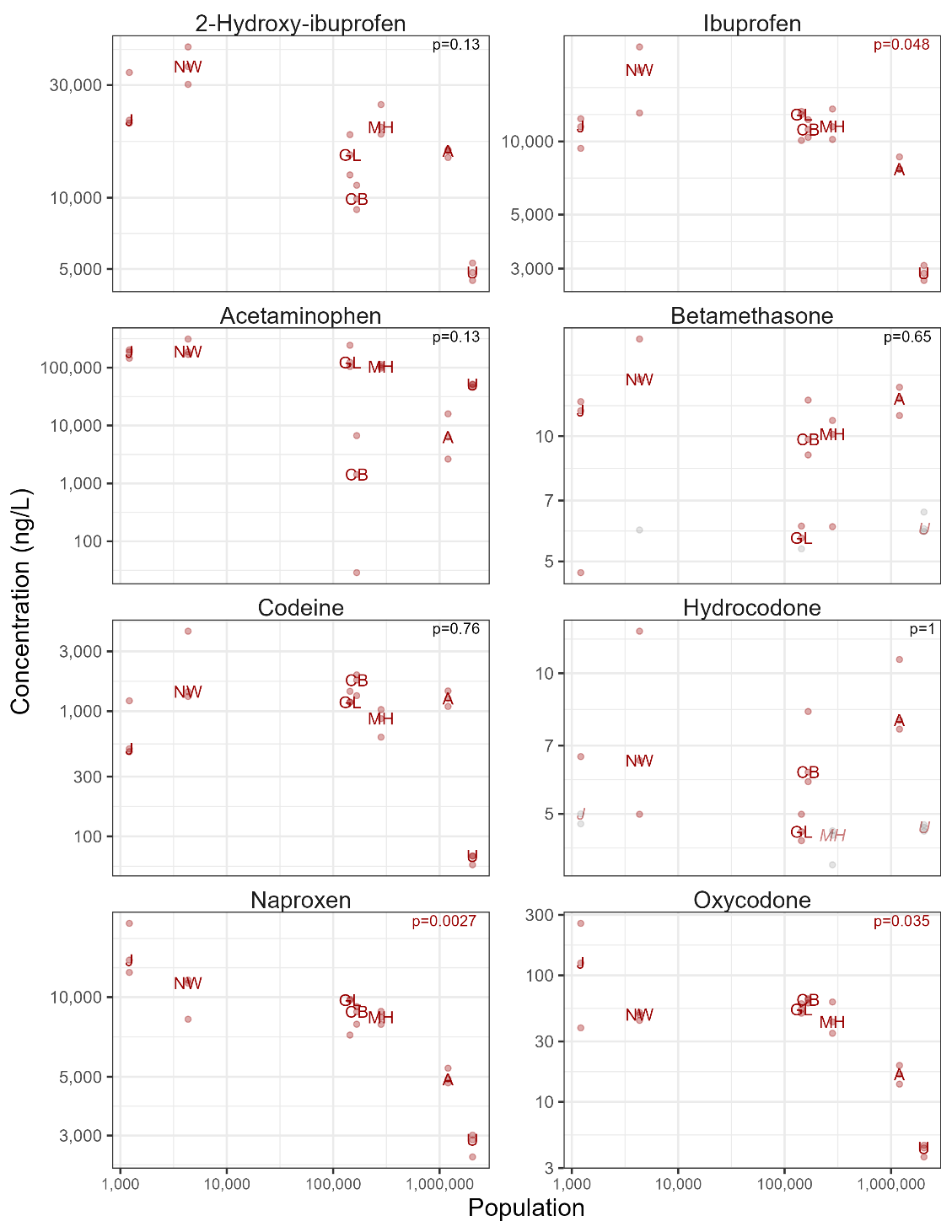
**

**
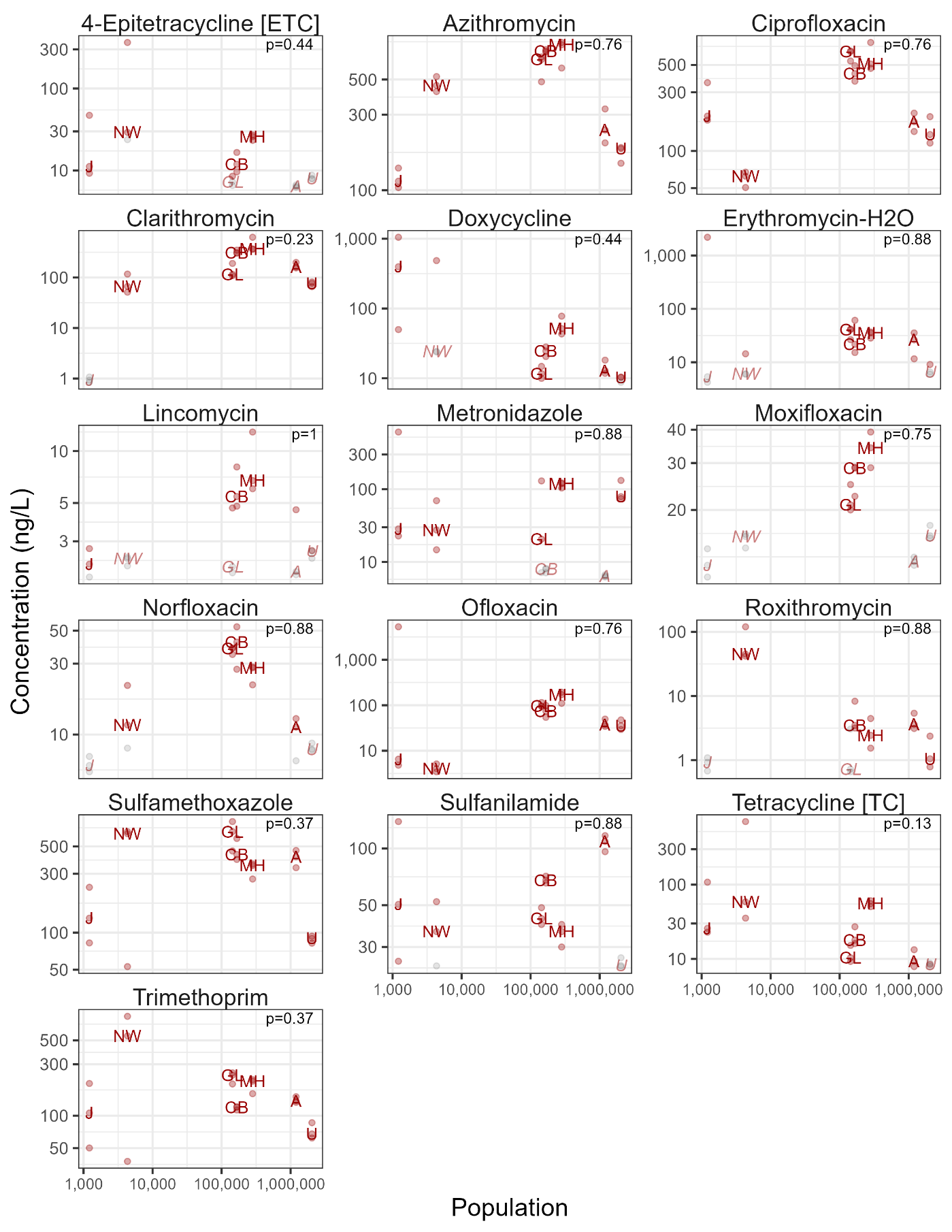
Fig. S2.1 b** (Antibiotic)

**Fig. S2.1 c** (Antihypertensive)


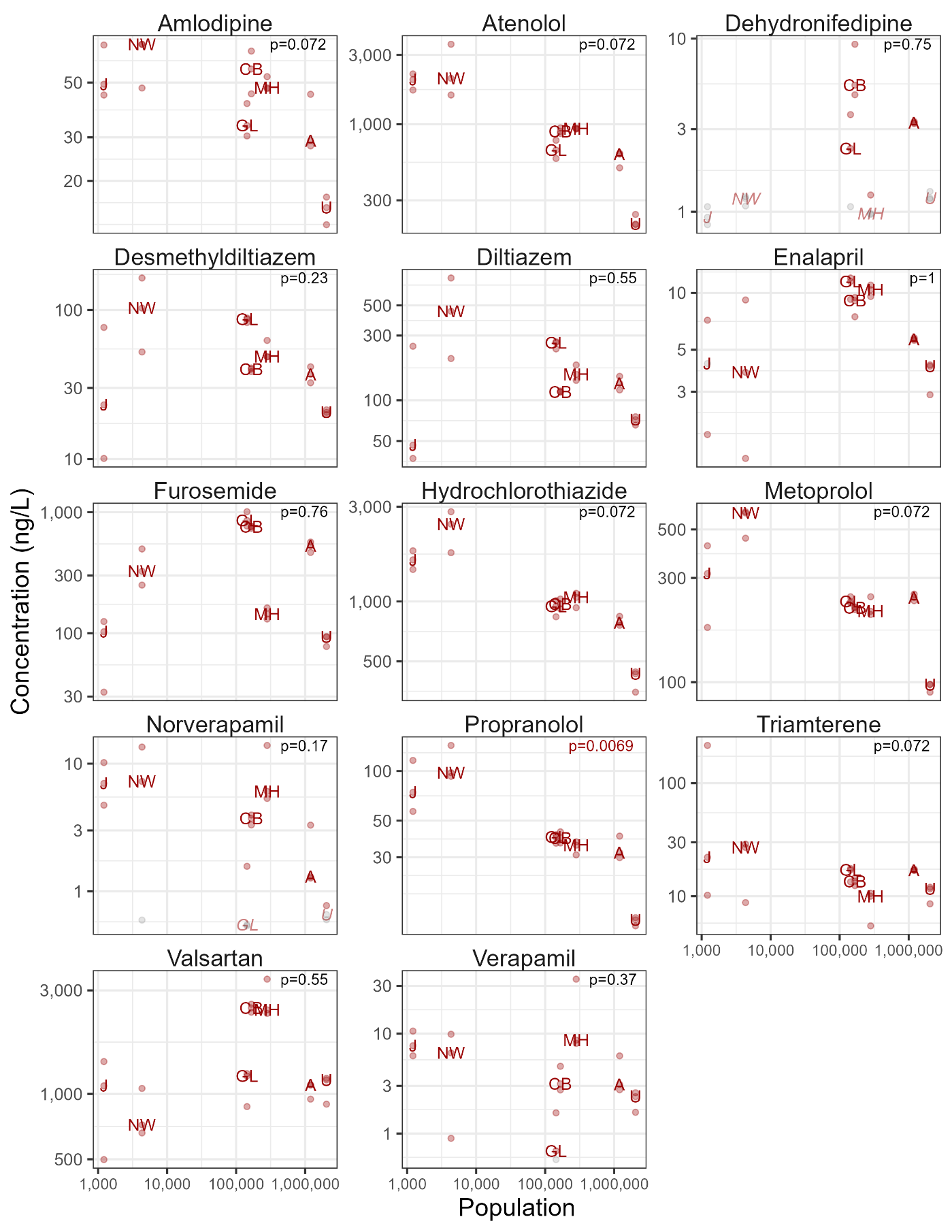


**Fig. S2.1 d** (Antimicrobial)

**
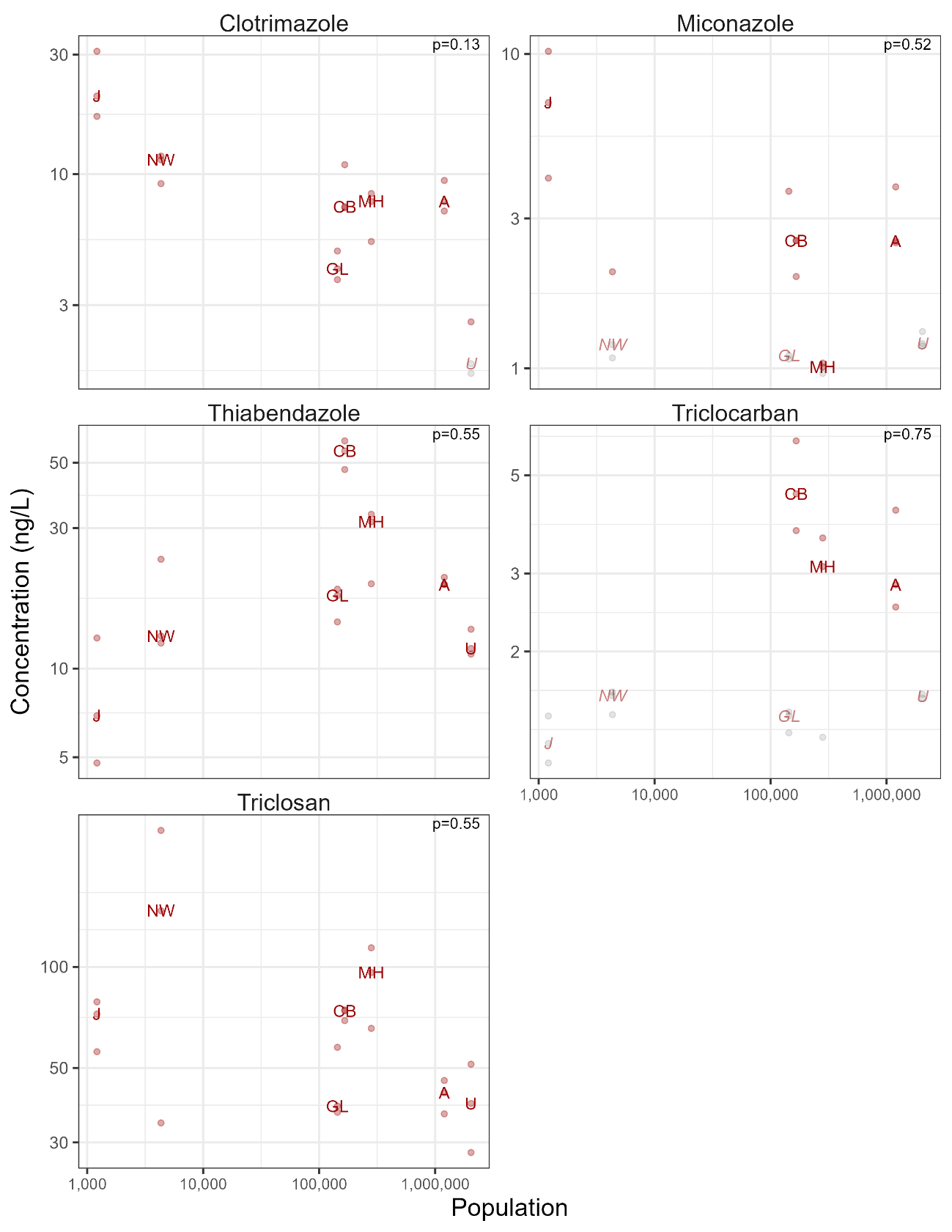
**

**Fig. S2.1 e** (Psychiatric)


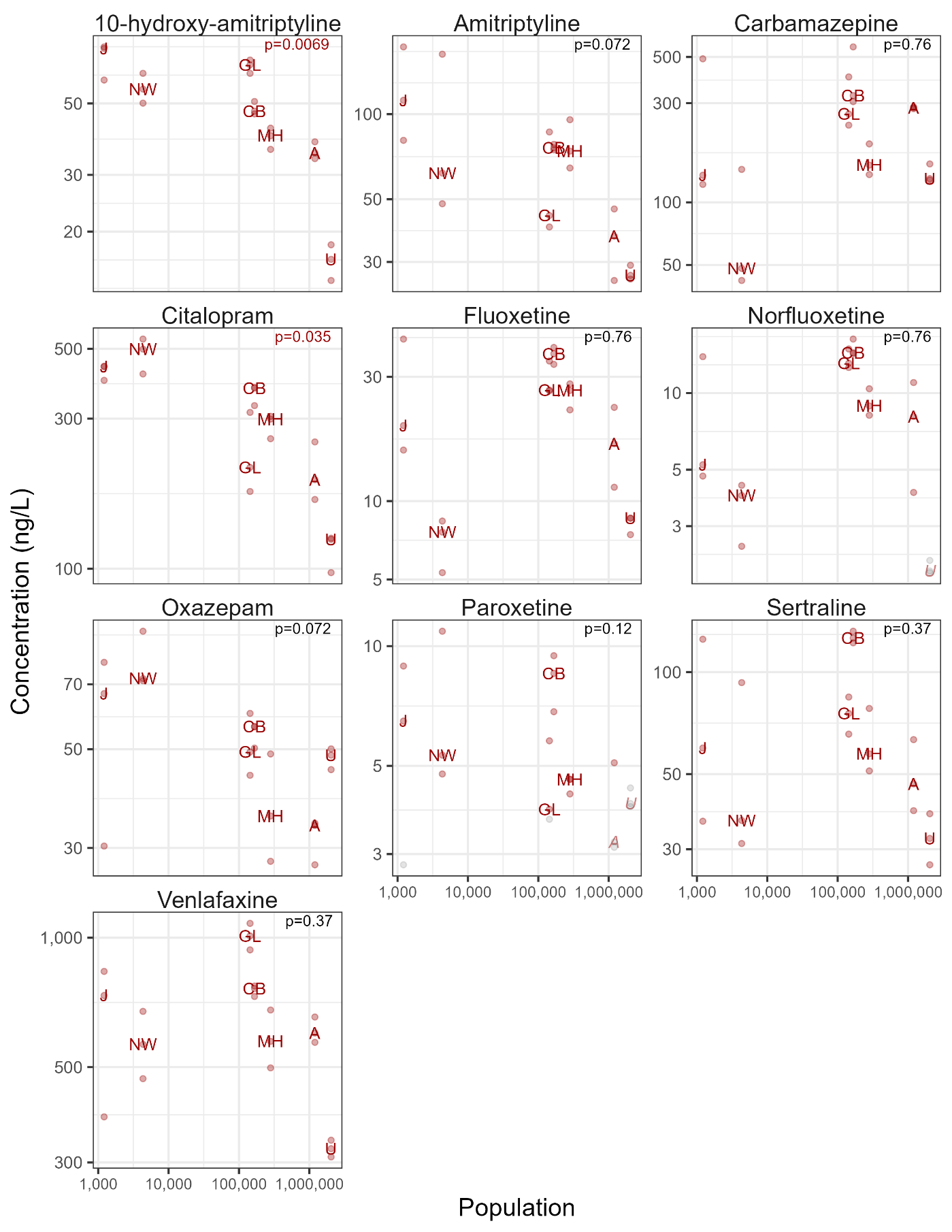


**Fig. S2.1 f** (Psychoactive stimulant)

**
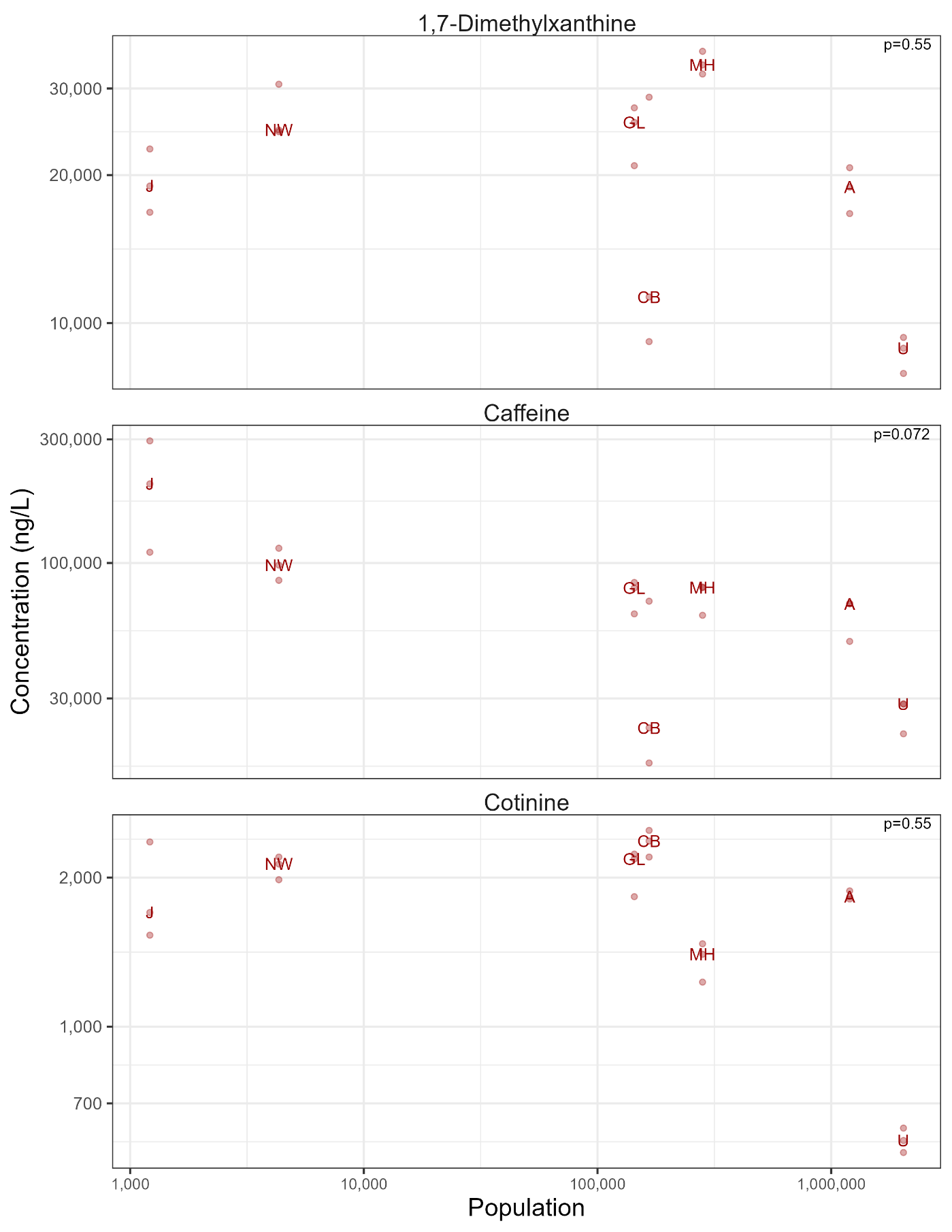
**

**
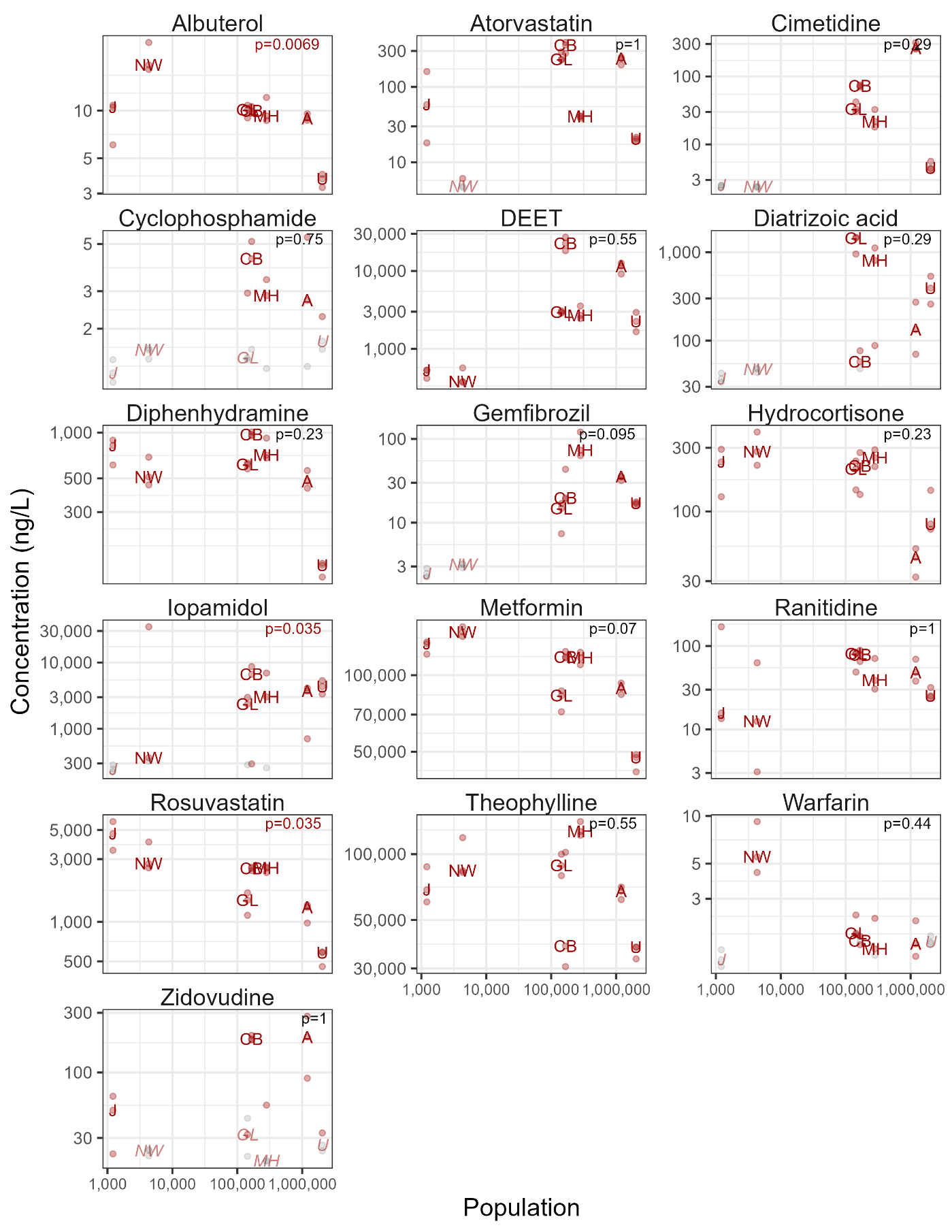
Fig. S2.1 g** (Other)

Fig. S2.2. Median calculated log solid-liquid distribution coefficients (K_d_) as a function of either log D_ow_ (pH = 7) for ionic PPCPs or log K_ow_ for non-ionic PPCPs. Data collected in 2022 were included in this figure.


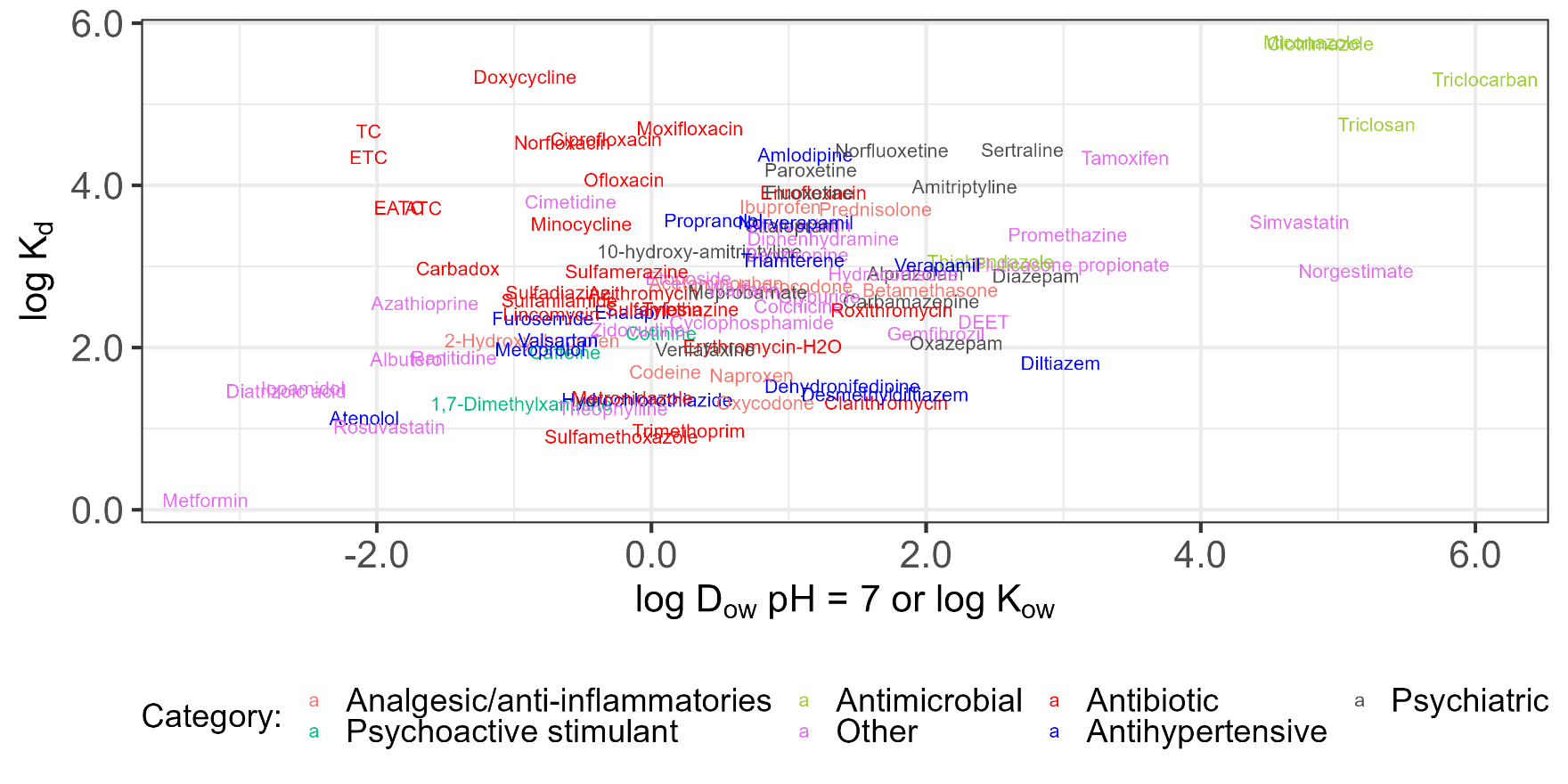


Fig. S2.3. Median percent removal as a function of median calculated log solid-liquid distribution coefficients (K_d_) for primary and secondary treatment facilities. Data collected in 2022 were included in this figure.


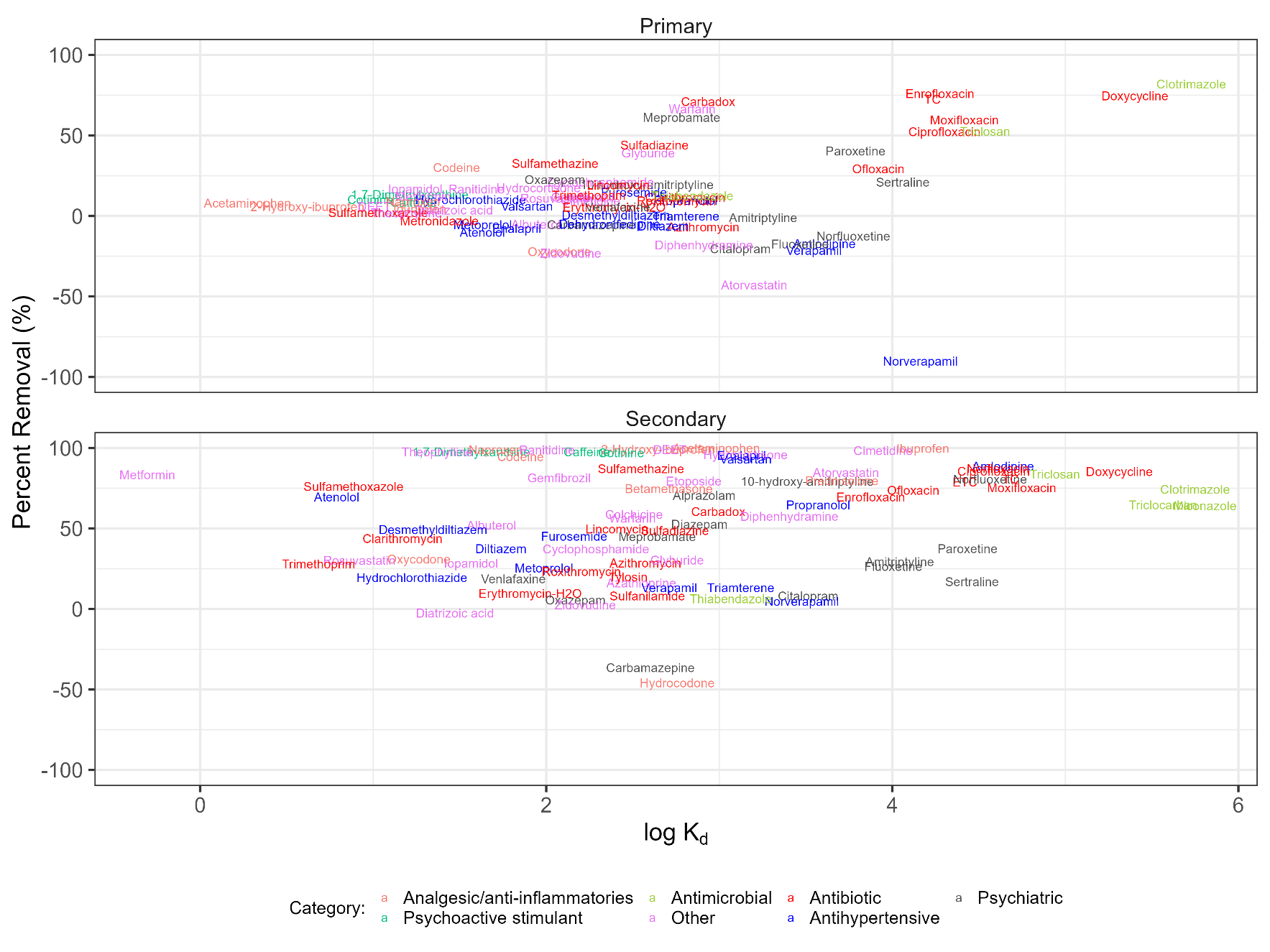
Note that two PPCPs, dehydronifedipine and metronidazole, that had low median removal of -188% and -405% respectively, were not included in the plot for secondary treatment plants.

Fig. S2.4 a-g. Comparison of PPCP concentrations in influent, effluent, and biosolids between 2010-13 and 2022.

The data points used in this figure represent the median PPCP concentration measured on three consecutive days at each WWTP and sampling event. The line within the boxes indicates median, the boxes indicate 25th and 75th percentiles, and the whiskers below and above the boxes indicate 10th and 90th percentiles. Dots indicate individual data points; red dots = detected concentrations, grey dots = concentrations below detection and plotted at the detection limit. PPCPs with medians above the detection limit in 50% of locations in at least one time group/media were included in this figure. Shaded rectangles indicate significant (p < 0.05) increase (pink) or decrease (blue) in PPCP concentrations between the two time periods. Potential factors (if any) that influence the time changes are indicated in black text with additional comments and details identified in Table S1.25. Note the log scale

**
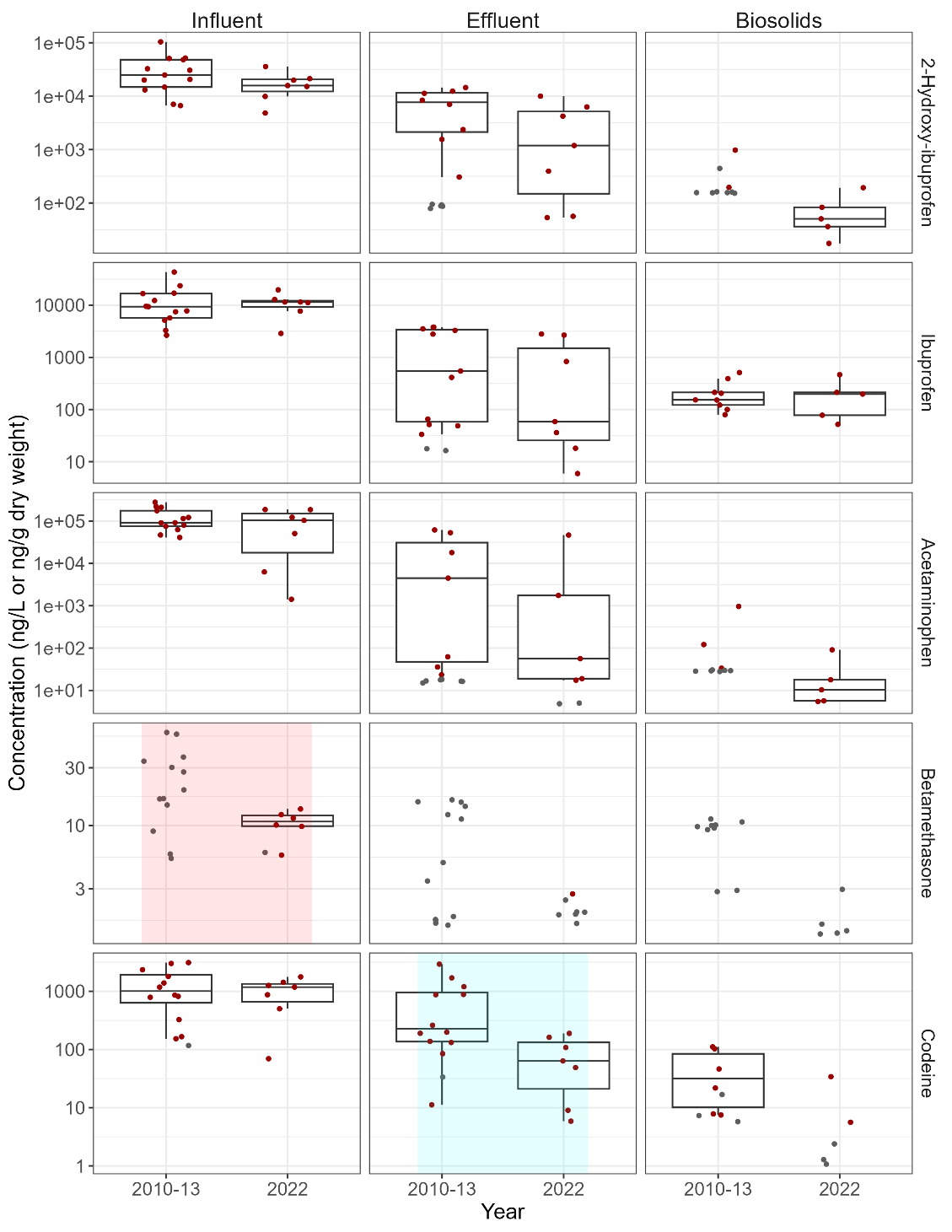
Fig. S2.4 a-1** (Analgesic/anti-inflammatories)

**Fig. S2.4 a-2** (Analgesic/anti-inflammatories continued)

**
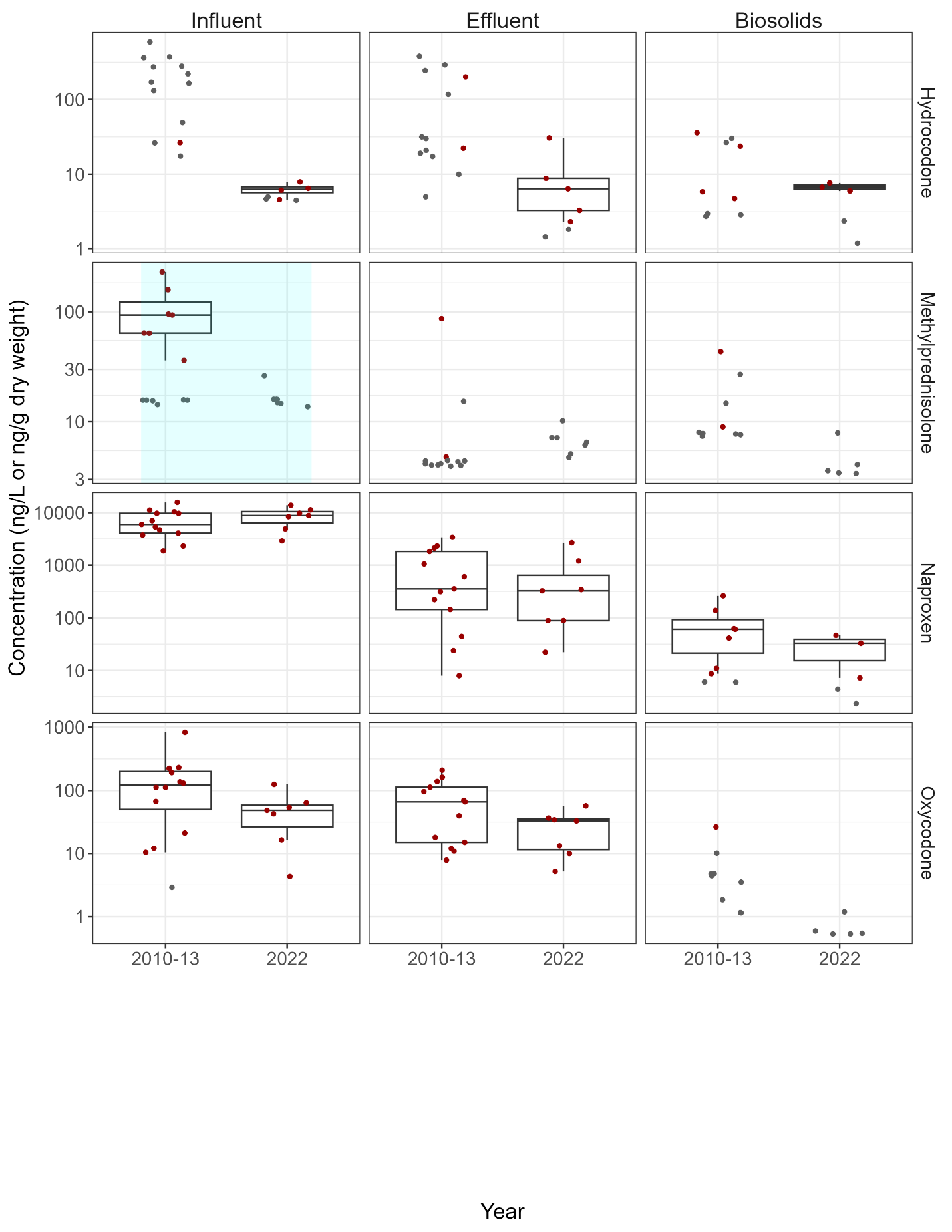
**


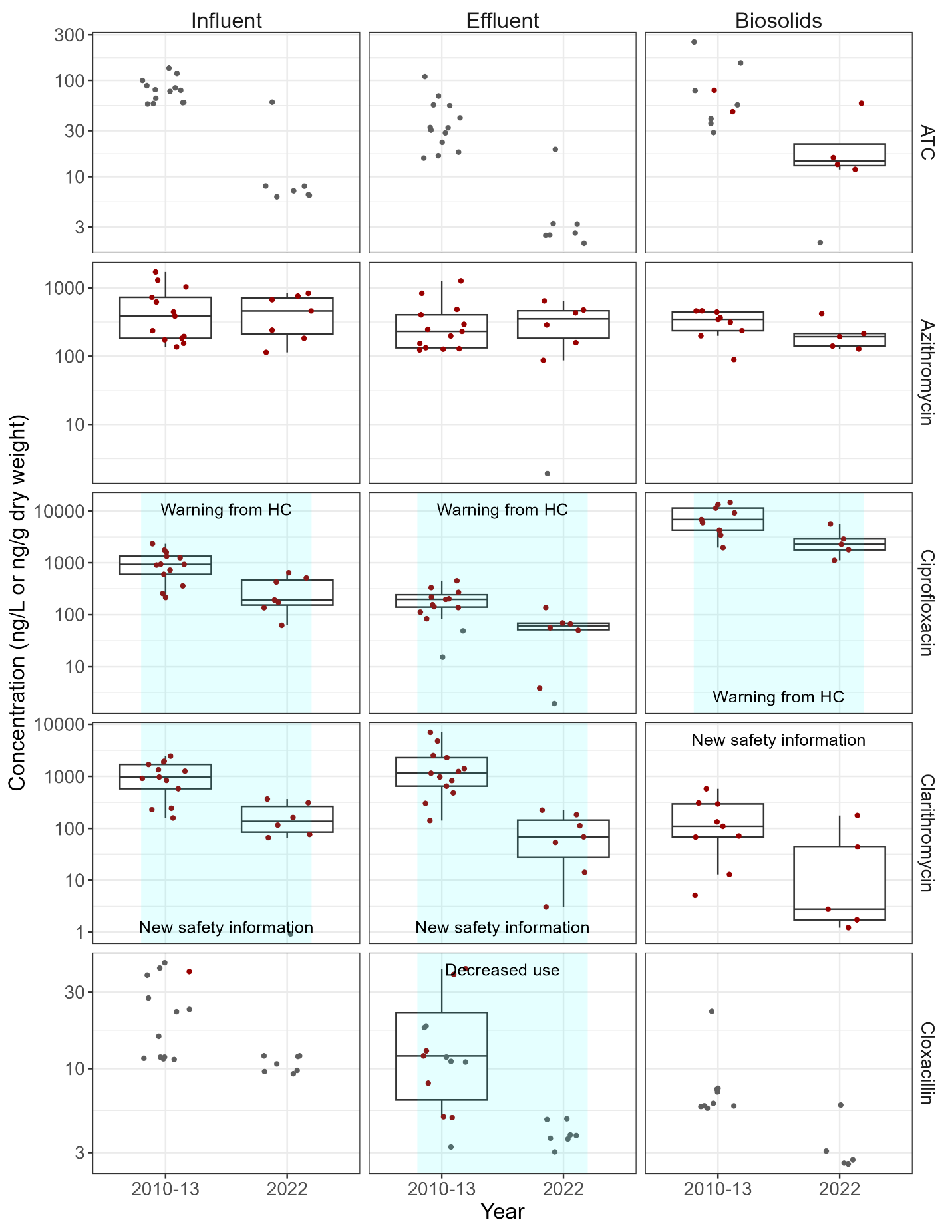
**Fig. S2.3 b-1** (Antibiotic)

**
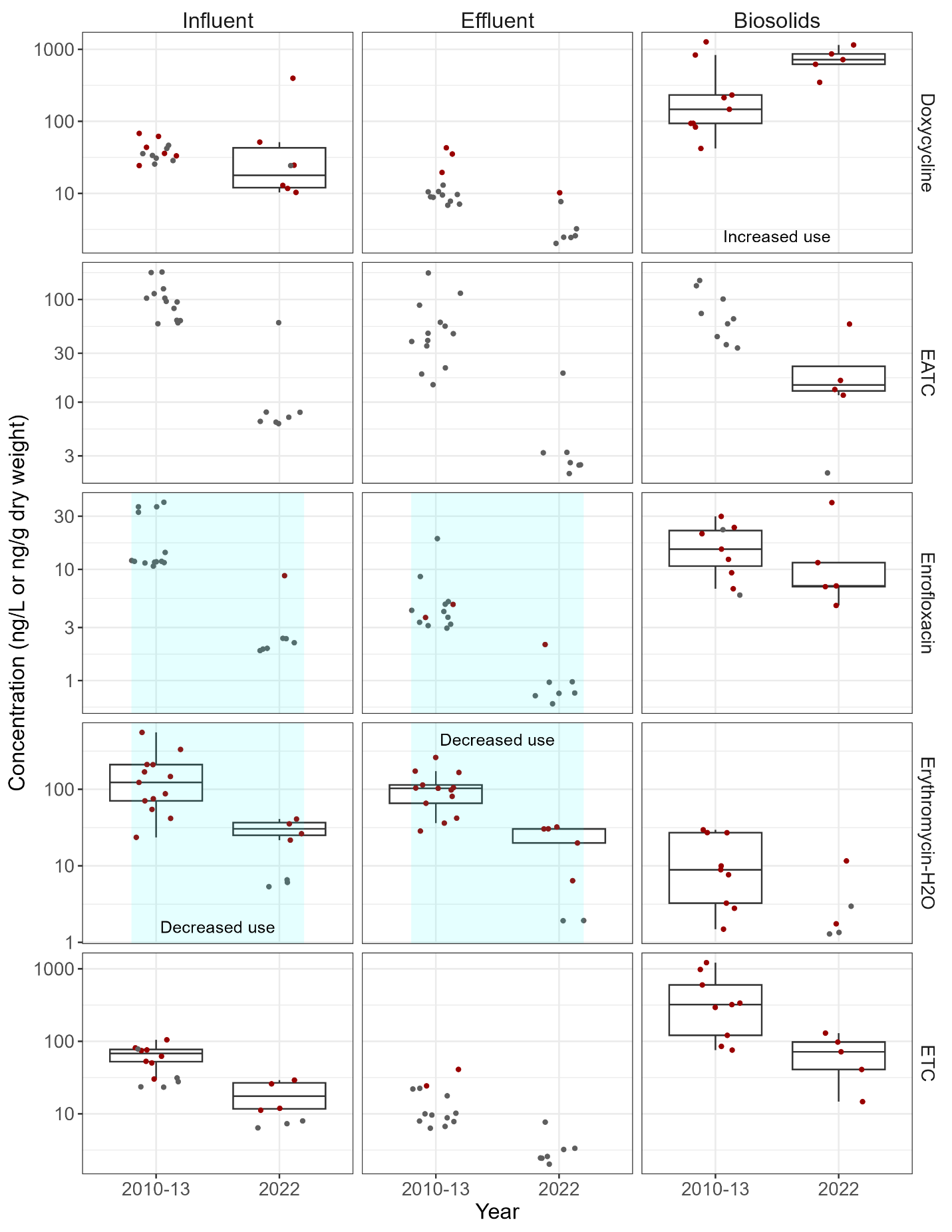
Fig. S2.4 b-2** (Antibiotic continued)

**
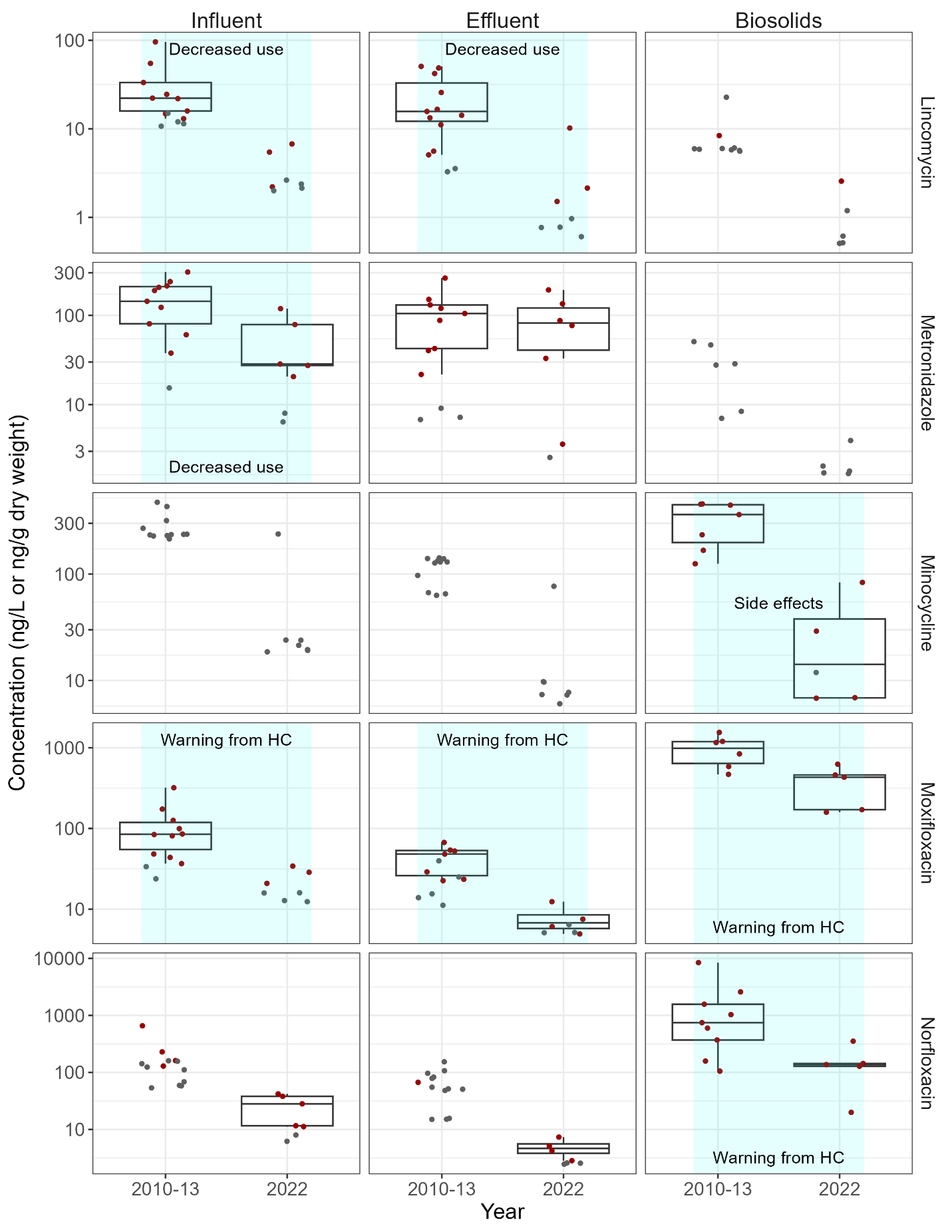
Fig. S2.4 b-3** (Antibiotic continued)

**
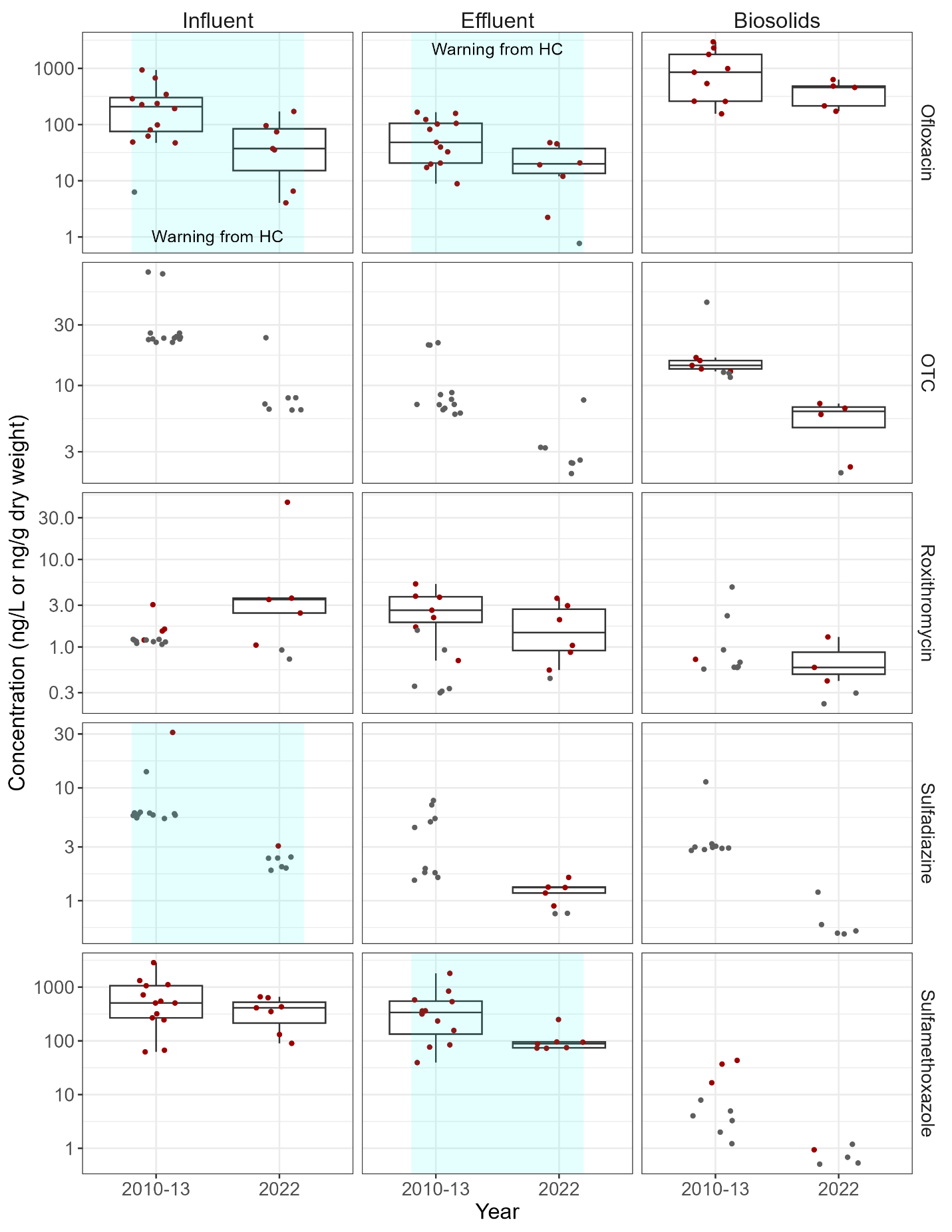
Fig. S2.4 b-4** (Antibiotic continued)

**Fig. S2.4 b-5** (Antibiotic continued)

**
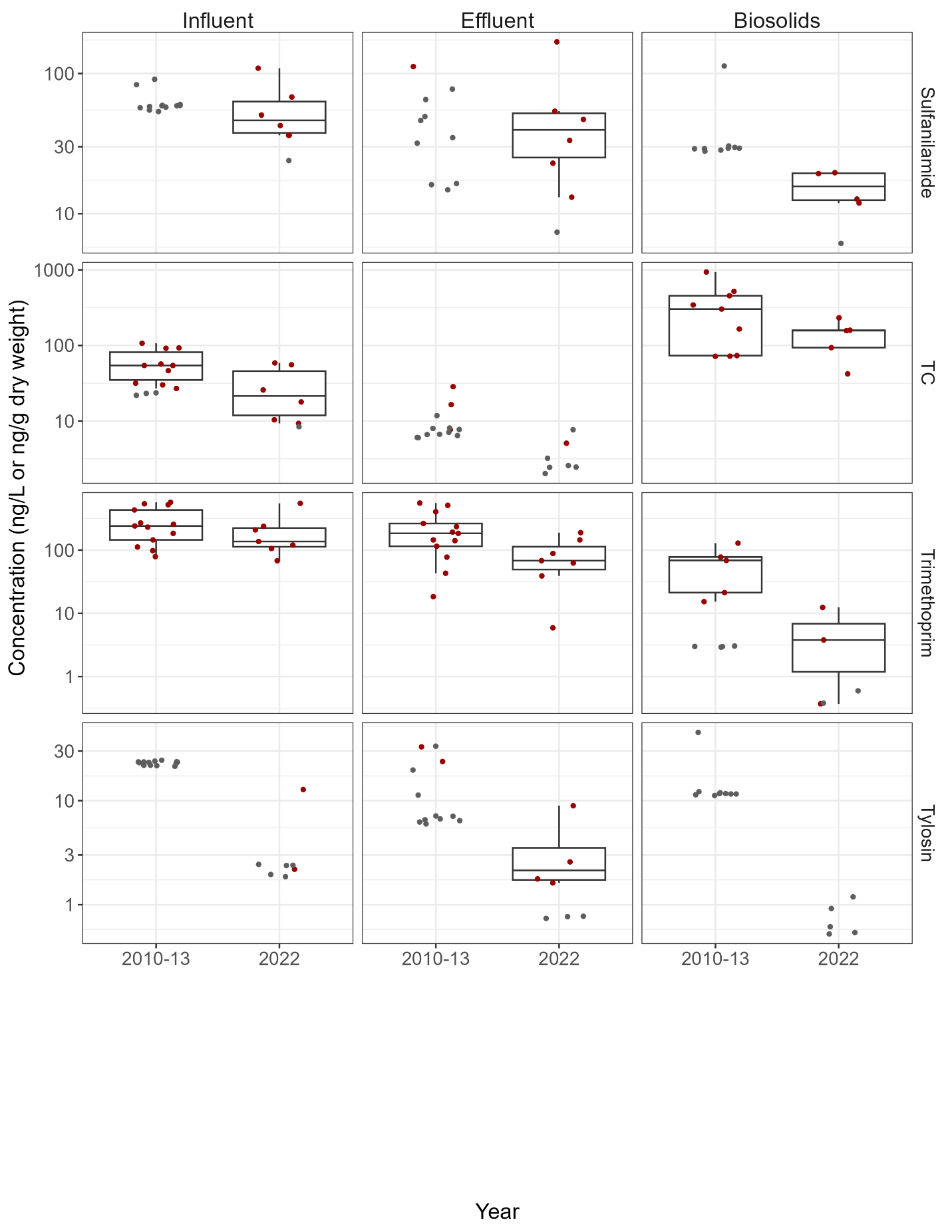
**

**
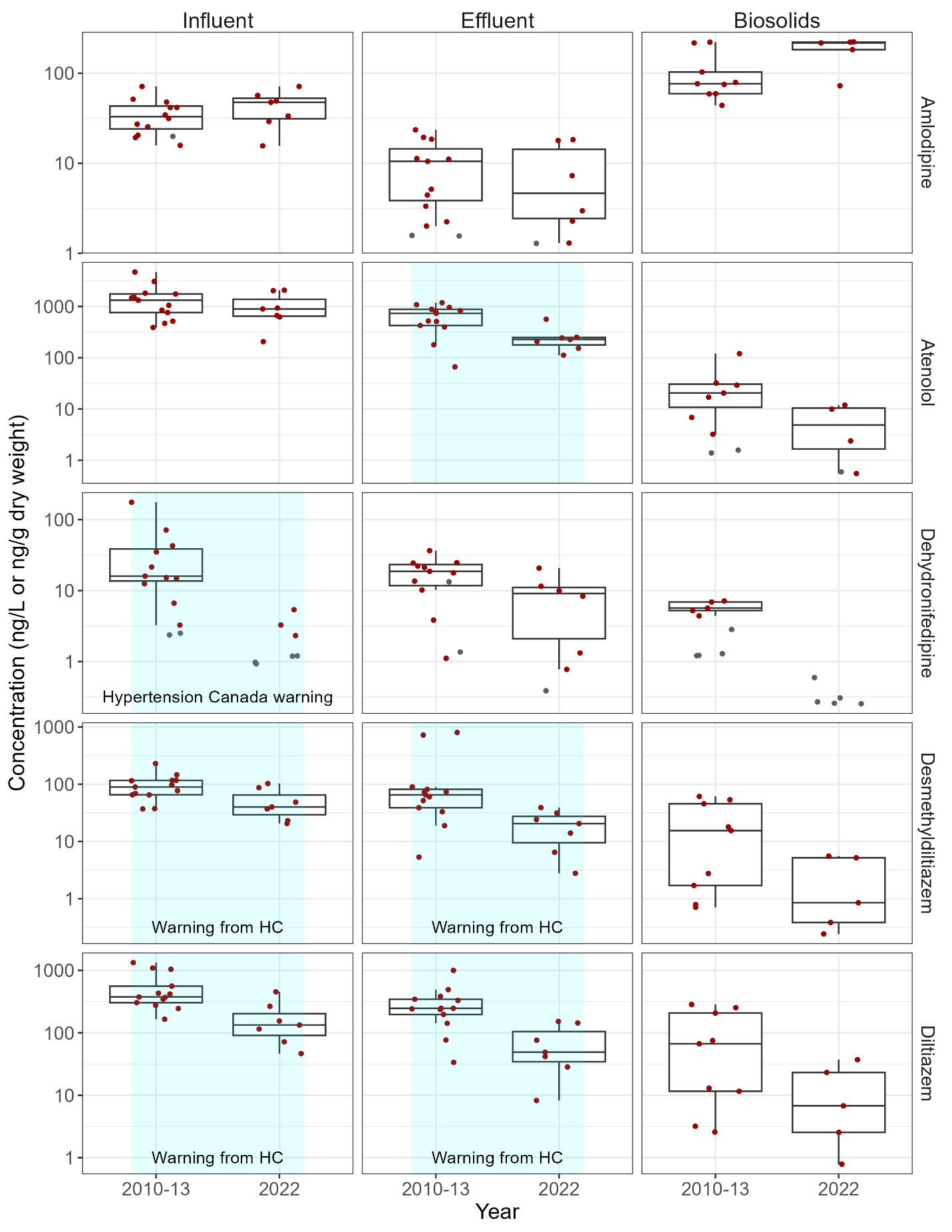
Fig. S2.4 c-1** (Antihypertensive)

**
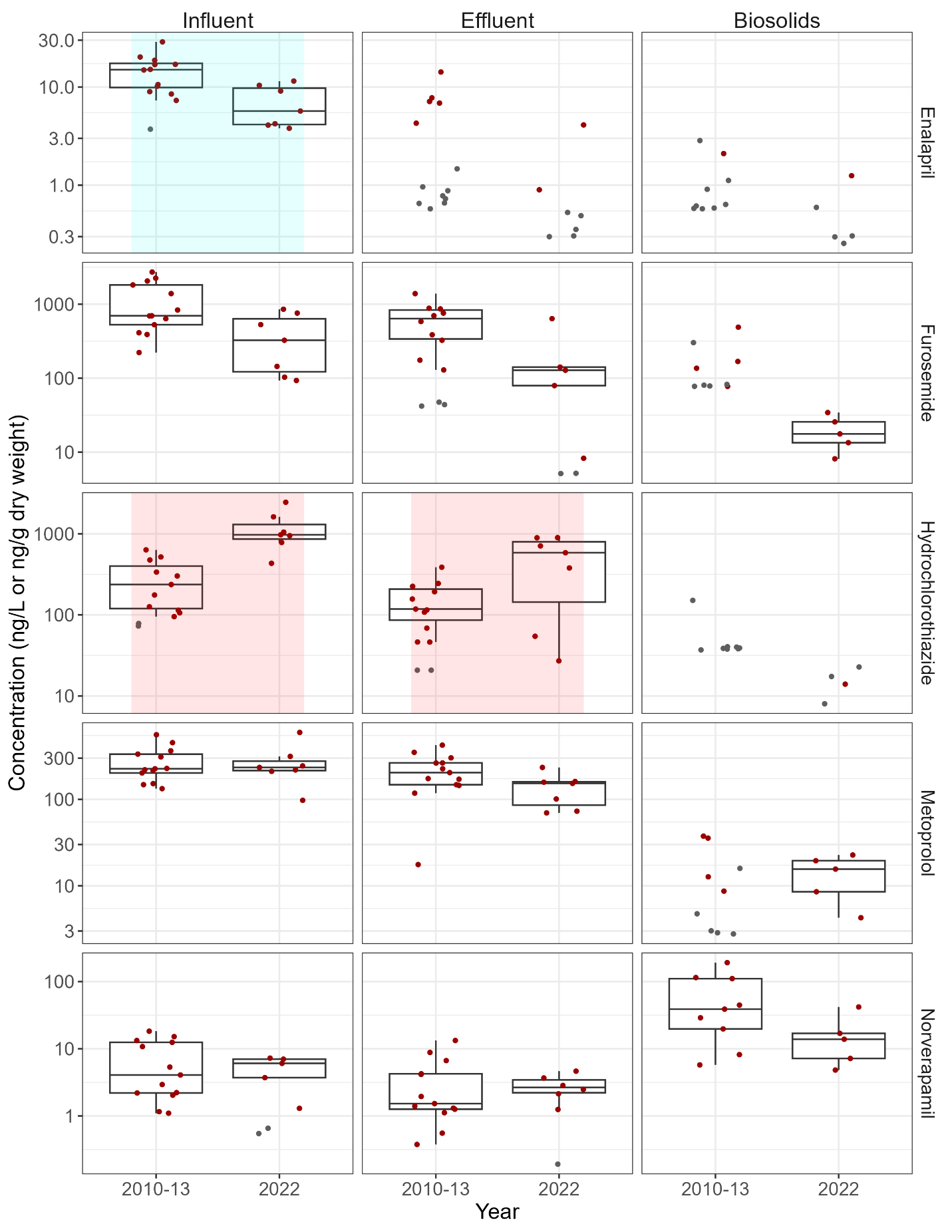
Fig. S2.4 c-2** (Antihypertensive continued)

**
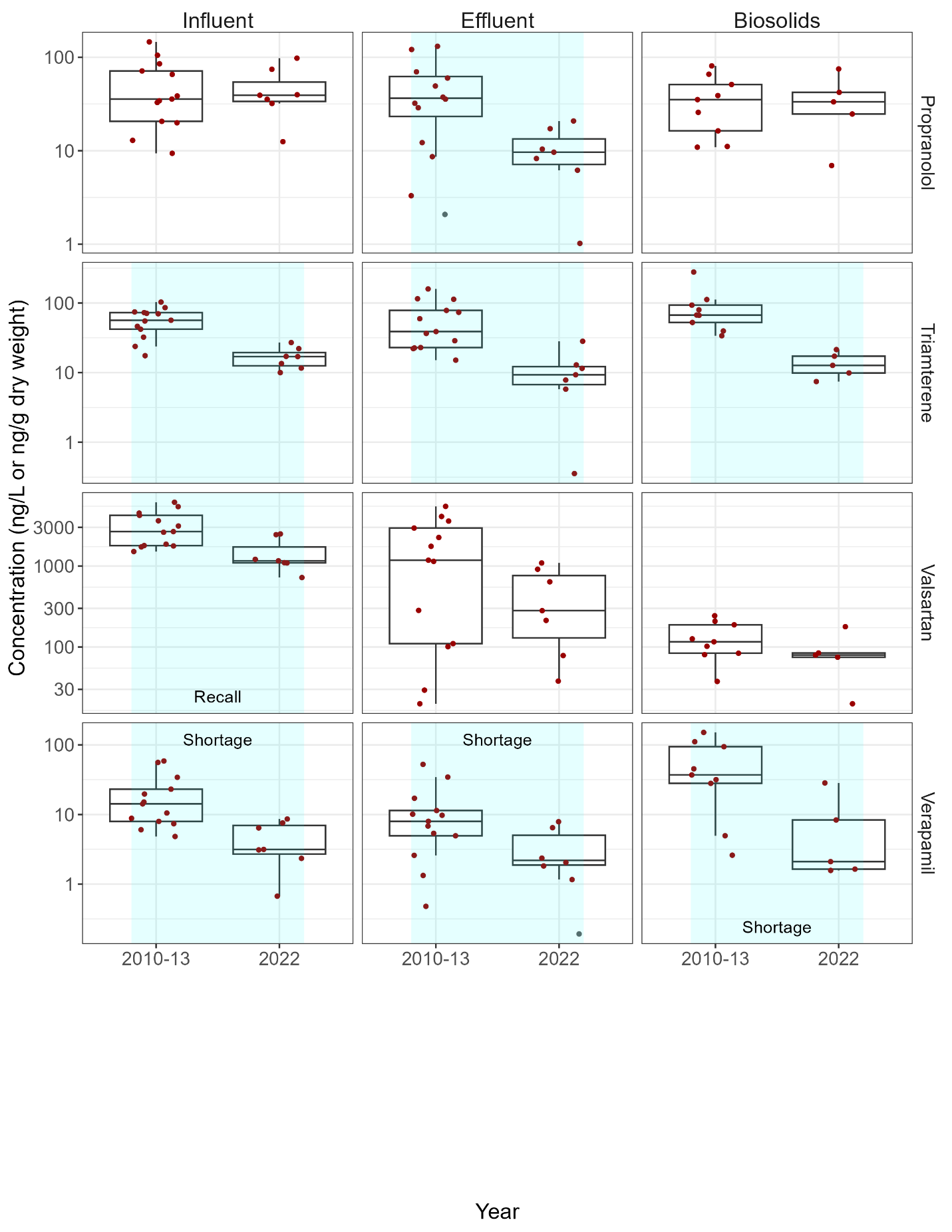
Fig. S2.4 c-3** (Antihypertensive continued)

**
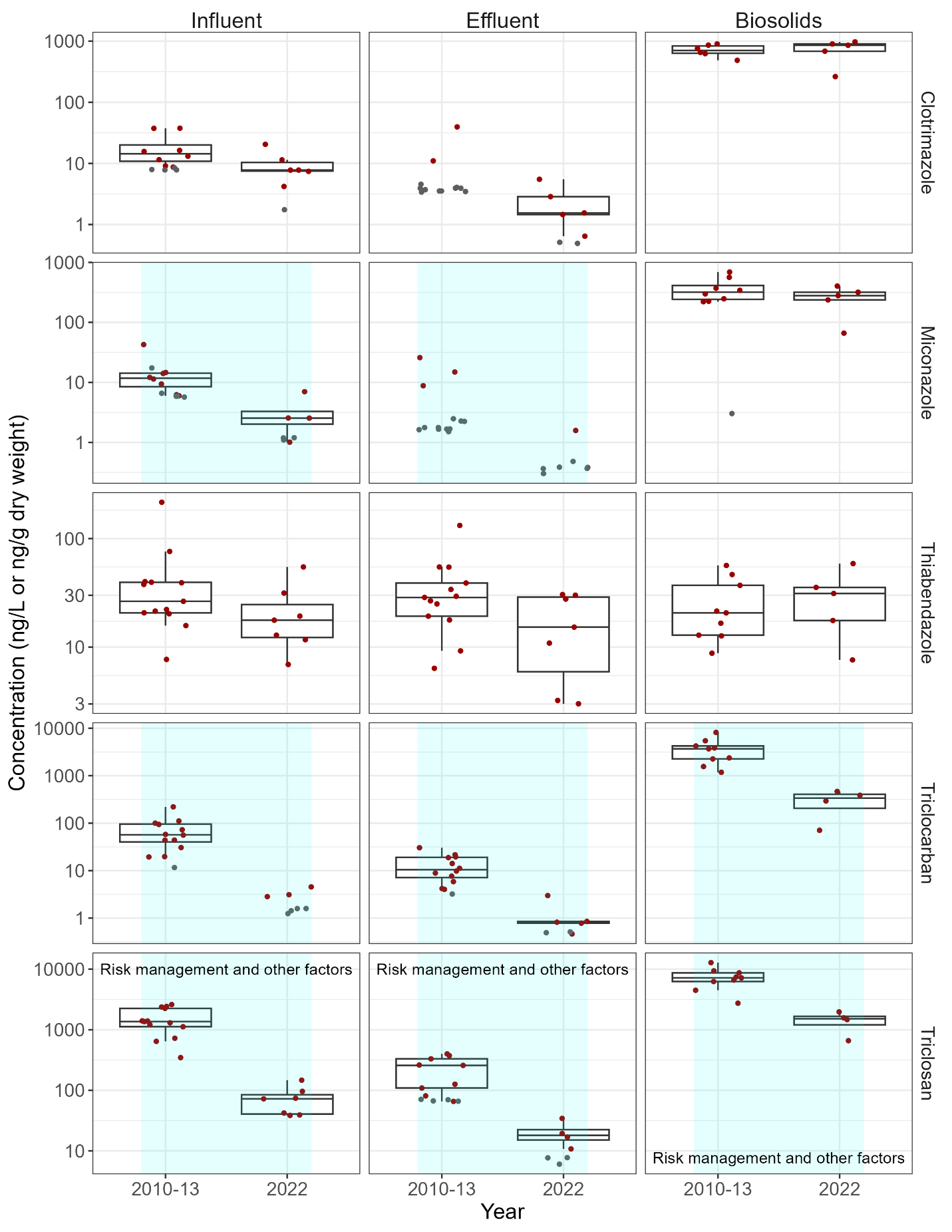
Fig. S2.4 d** (Antimicrobial)

**
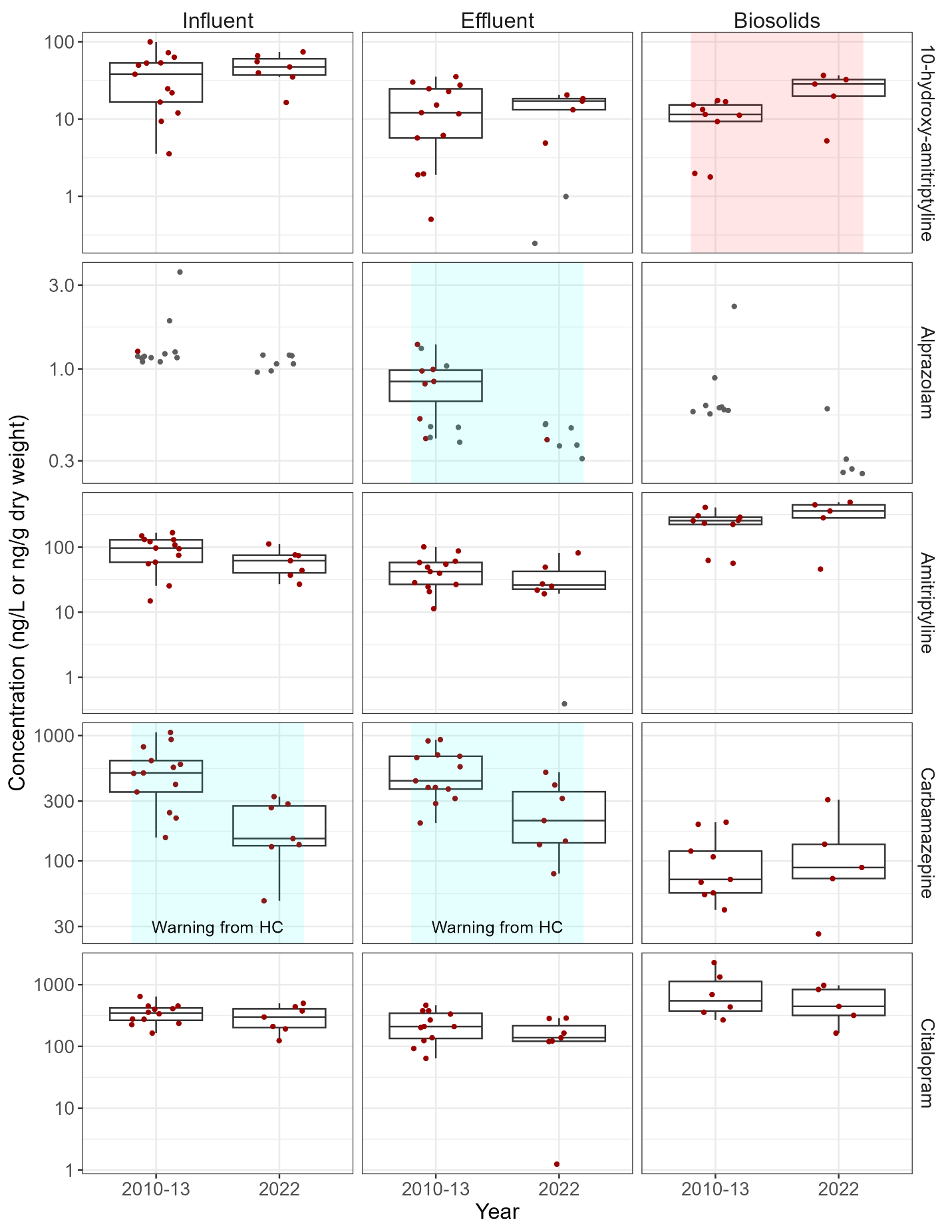
Fig. S2.4 e-1** (Psychiatric)

**Fig. S2.4 e-2** (Psychiatric continued)

**2** (Psychiatric continued)

**
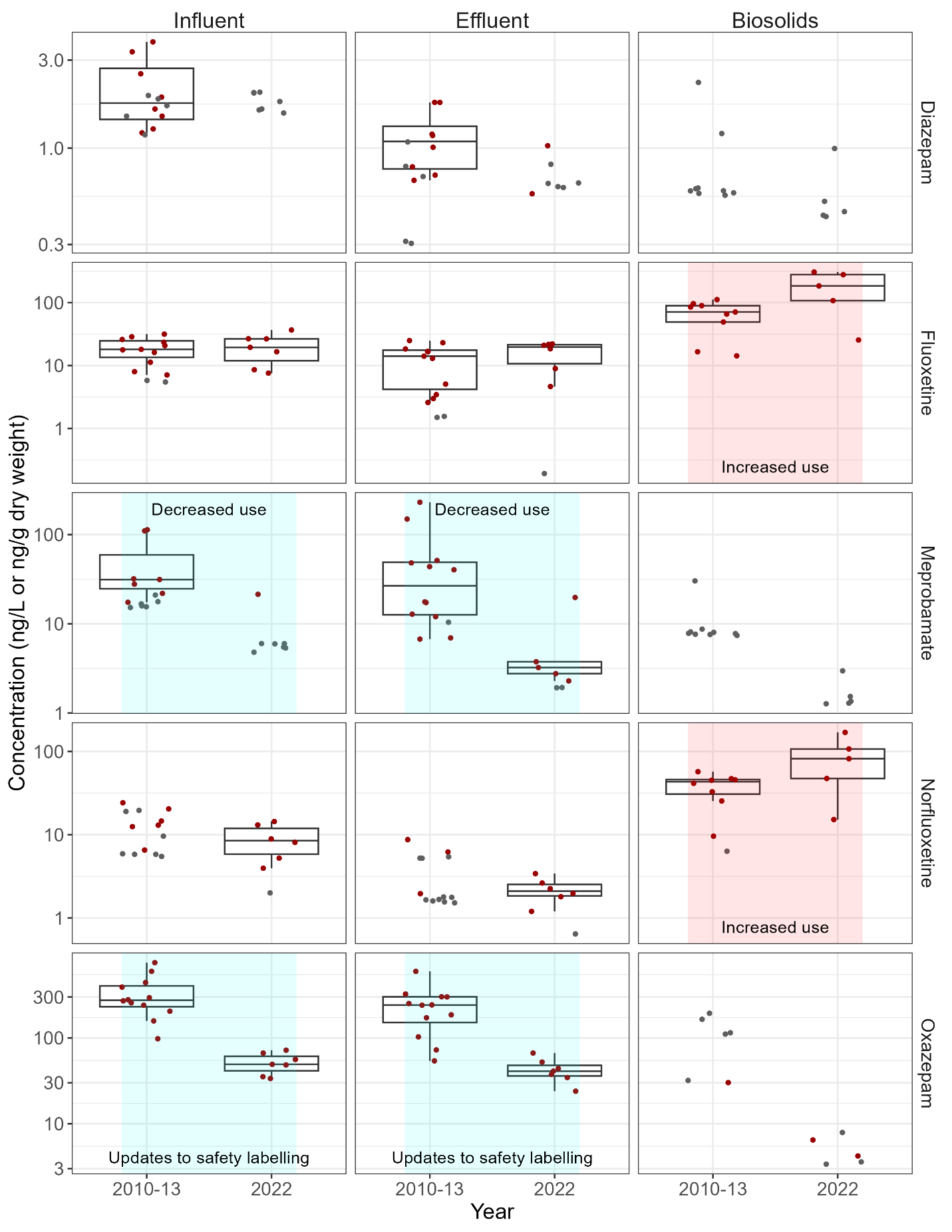
**

**
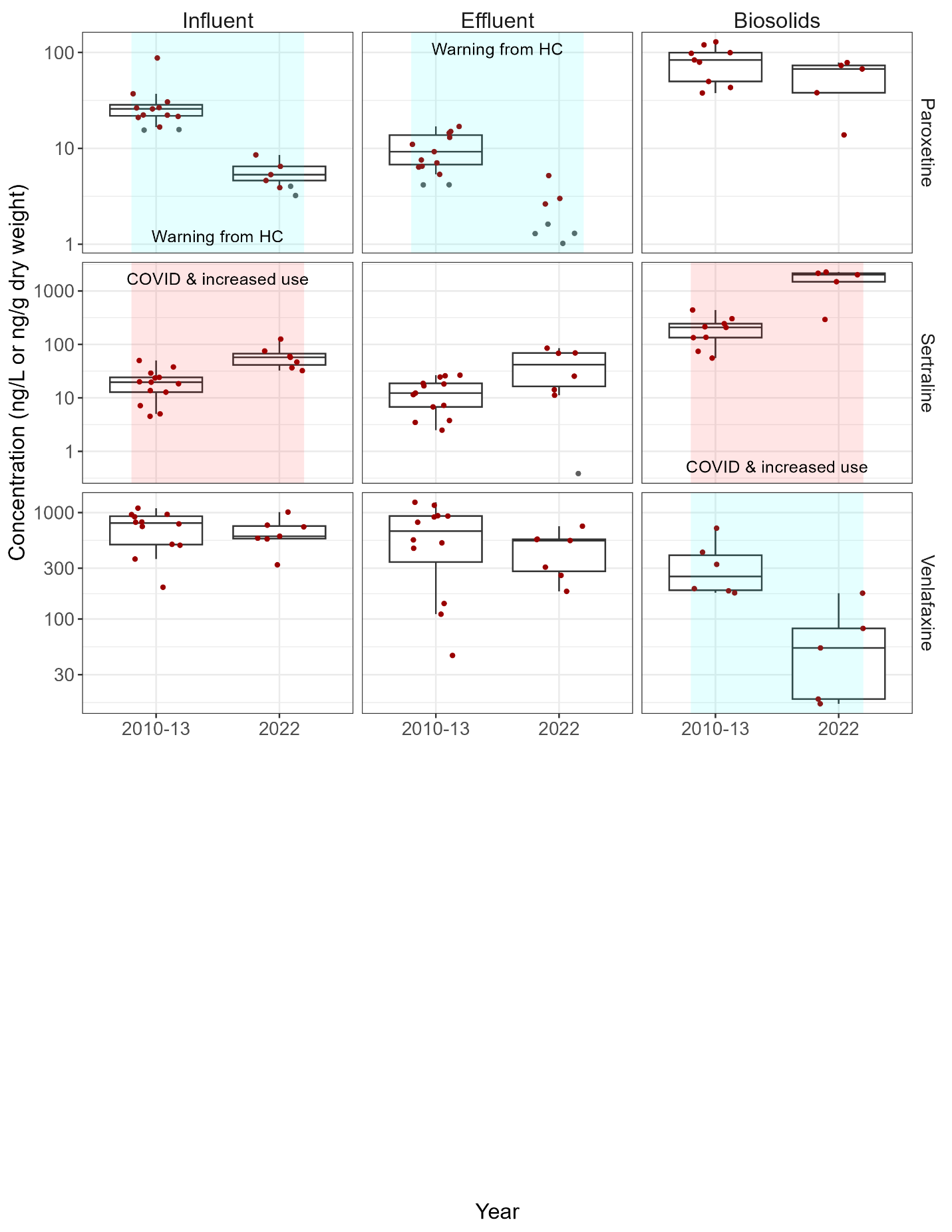
Fig. S2.4 e-3** (Psychiatric continued)

**
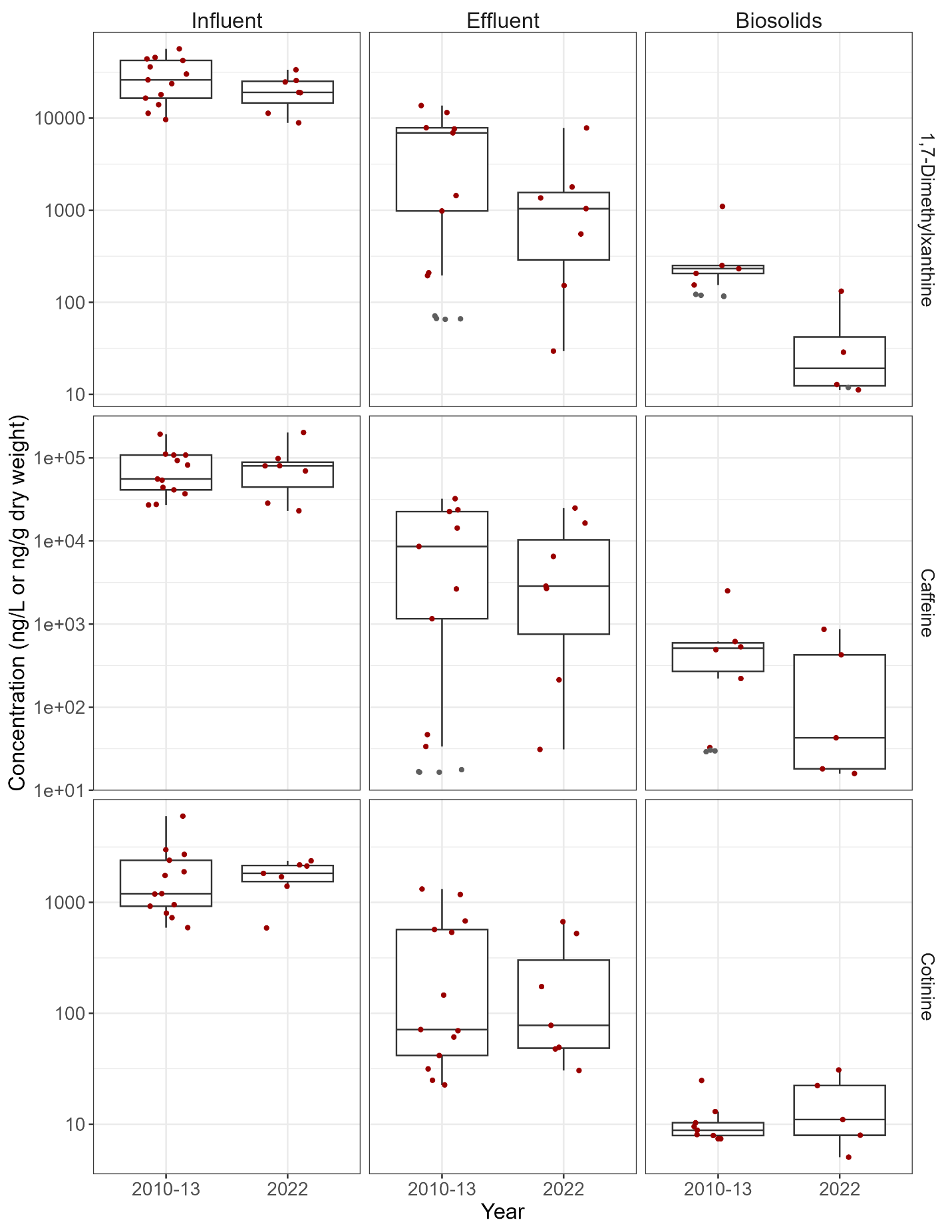
Fig. S2.4 f** (Psychoactive stimulant)

**
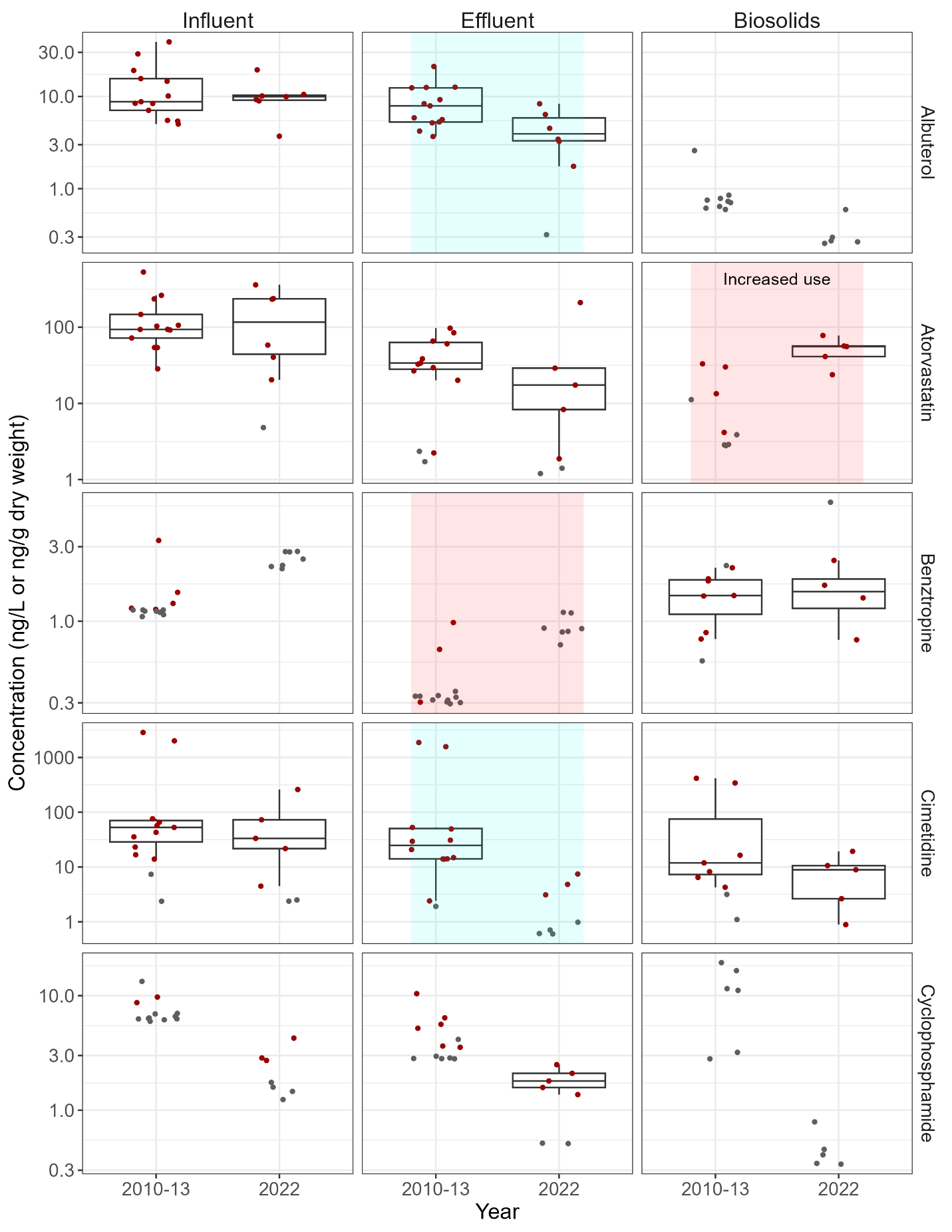
Fig. S2.4 g-1** (Other)

**
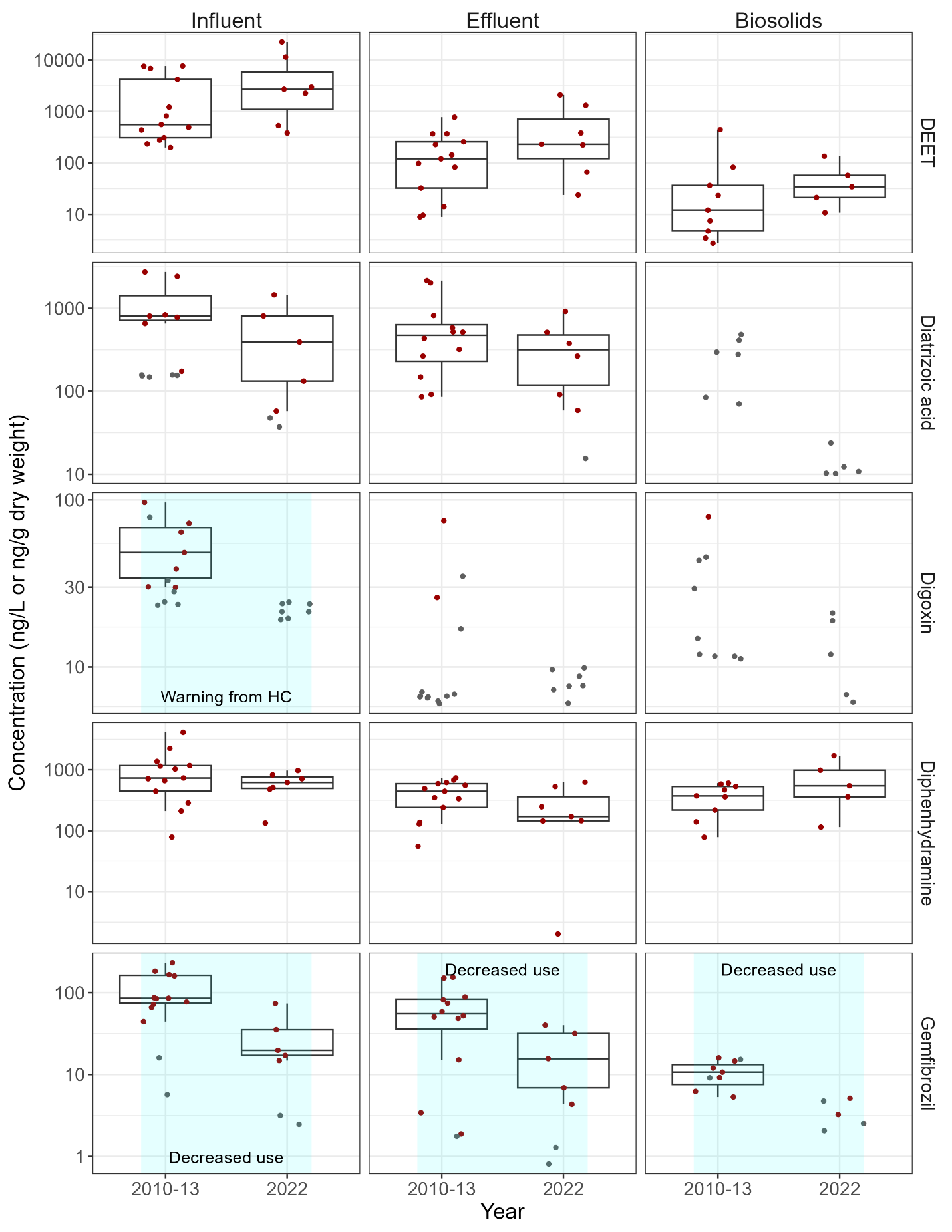
Fig. S2.4 g-2** (Other continued)

**
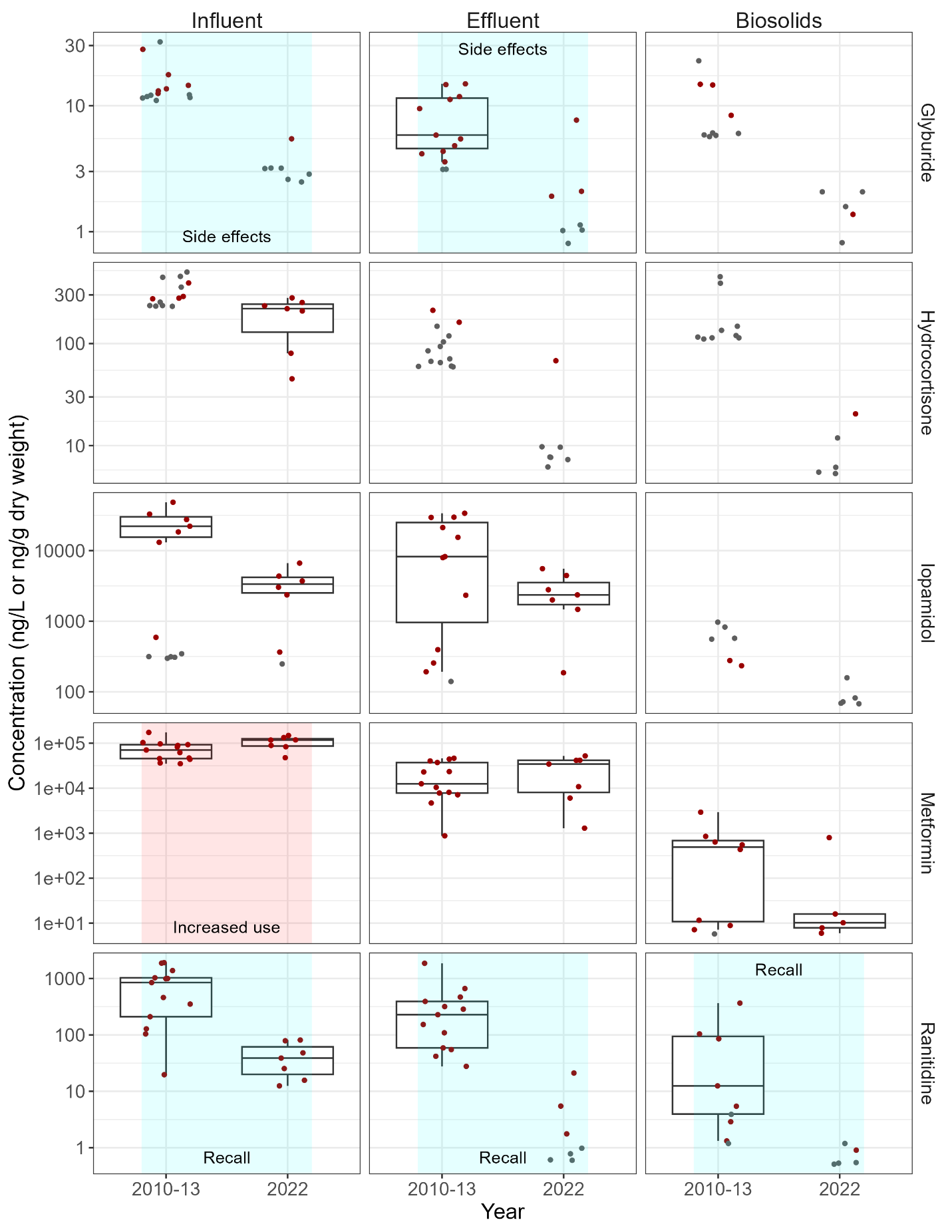
Fig. S2.4 g-3** (Other continued)

**
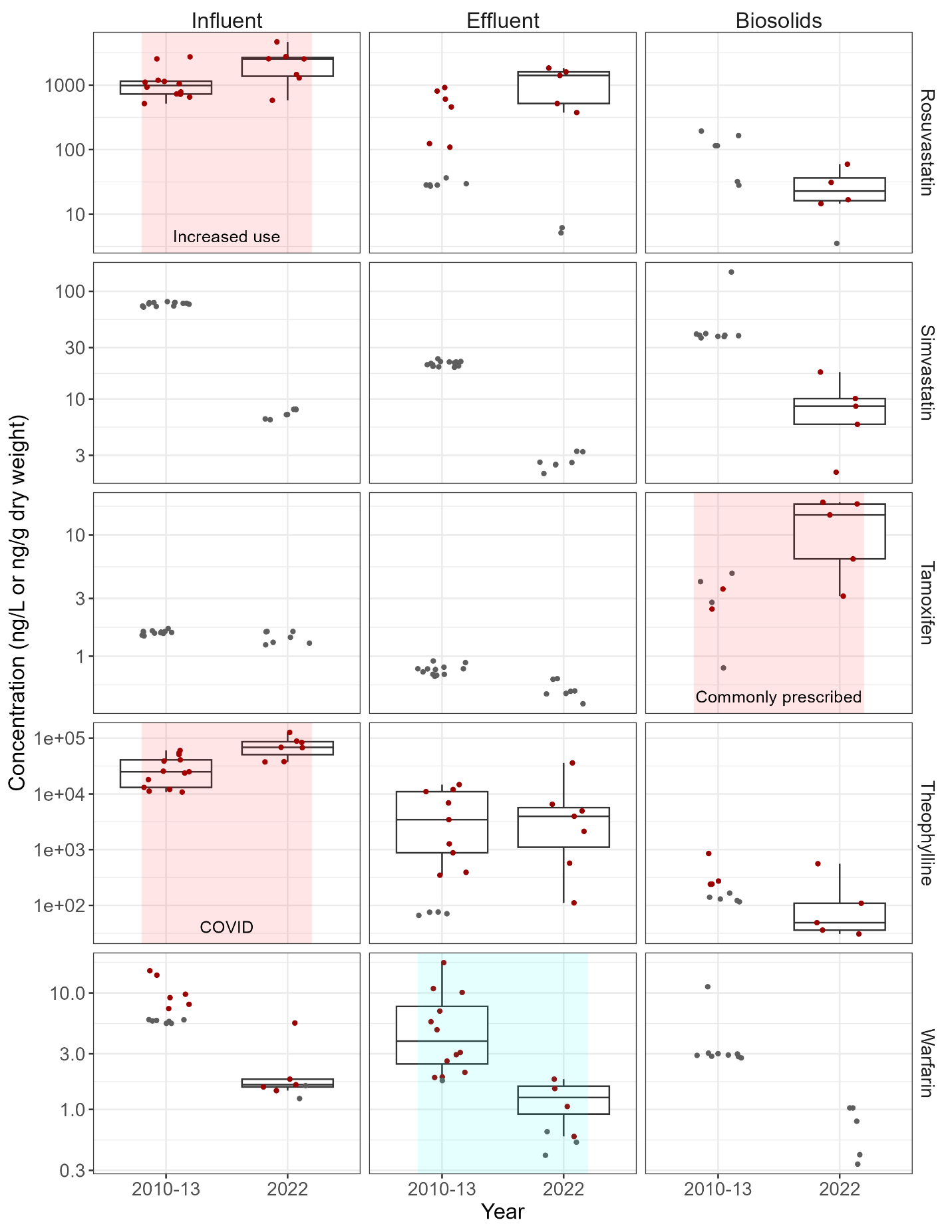
Fig. S2.4 g-4** (Other continued)

**Fig. S2.4 g-5** (Other continued)

**
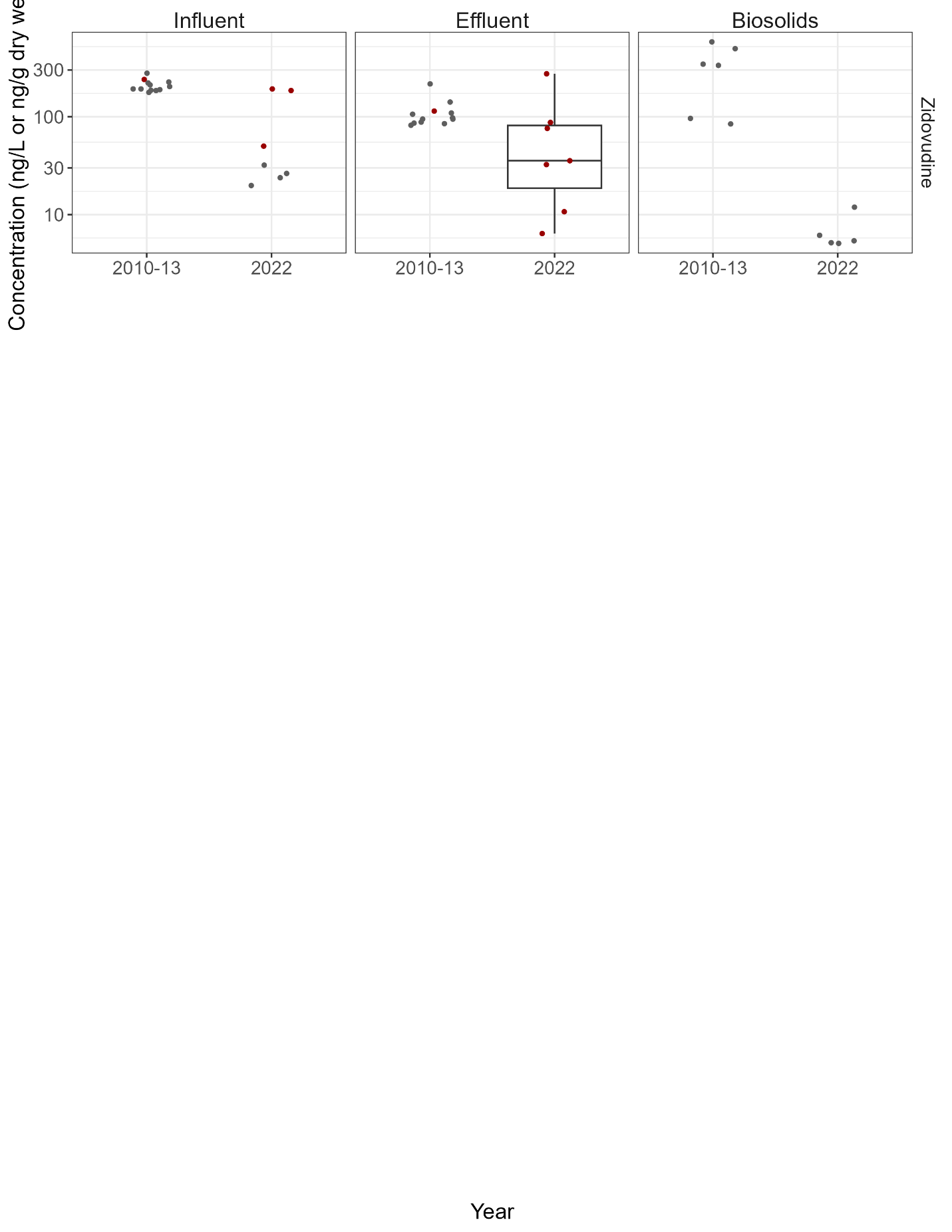
**

# References

American Public Health Association (2012) Standard methods for the examination of water and wastewater, 22nd edn. American Public Health Association, Washington, DC, USA

Government of Canada (2024) Chemicals Management Plan wastewater monitoring program. https://data-donnees.az.ec.gc.ca/data/substances/monitor/chemicals-management-plan-wastewater-monitoring-program/?lang=en. Accessed 25 Jun 2024

Metcalf & Eddy Inc (2003) Wastewater engineering, treatment and reuse. McGraw-Hill, New York, NY, USA

USEPA (2007) Method 1694: Pharmaceuticals and personal care products in water, soil, sediment and biosolids by HPLC/MS/MS. https://www.epa.gov/sites/default/files/2015-10/documents/method_1694_2007.pdf. Accessed 29 May 2024
